# Supplementary material for: Nanopore Deep Sequencing as a Tool to Characterize and Quantify Aberrant Splicing Caused by Variants in Inherited Retinal Dystrophy Genes
Source: Int J Mol Sci. 2024 Sep 3;25(17):9569. doi: 10.3390/ijms25179569 (PMC11395040; doi:10.3390/ijms25179569)

Figure S3. Gel electrophoresis from the reference and variant minigenes for each variant. Abbreviations: WT, wildtype (reference) minigene; MT, mutant (variant) minigene.

RHO_minigene_ABCA4_int4-6

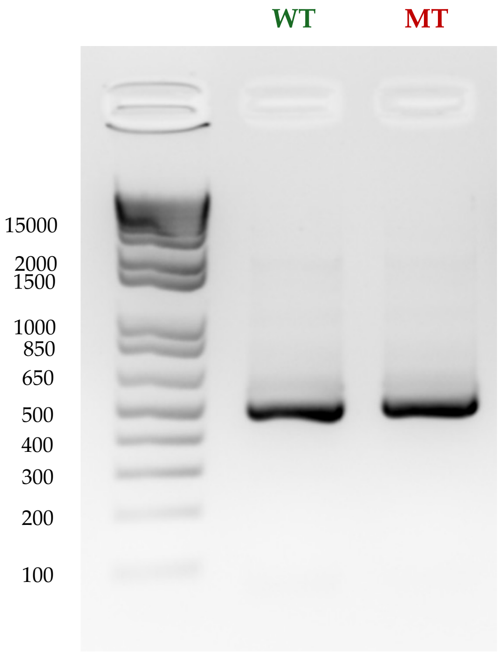

RHO_minigene_ABCA4_int38-41

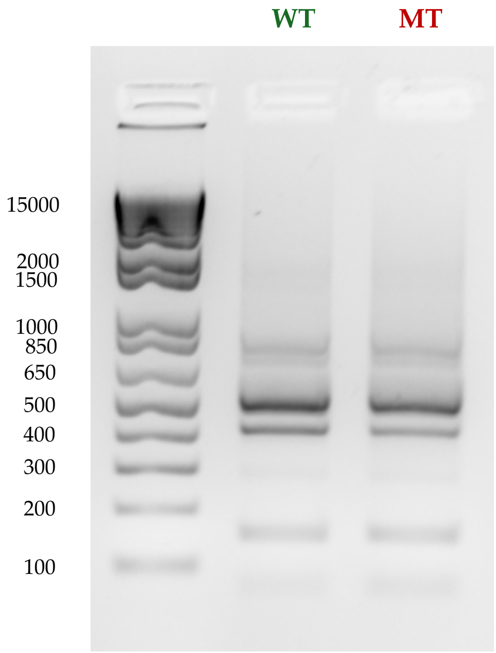

RHO_minigene_ATF6_int8-9

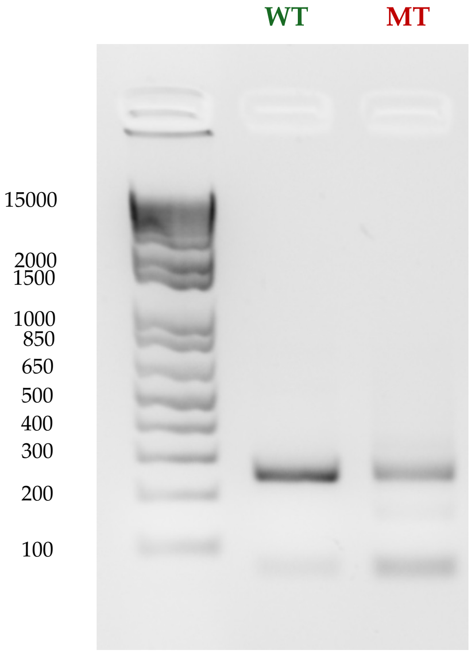

ATF6_minigene_ex1-2-9

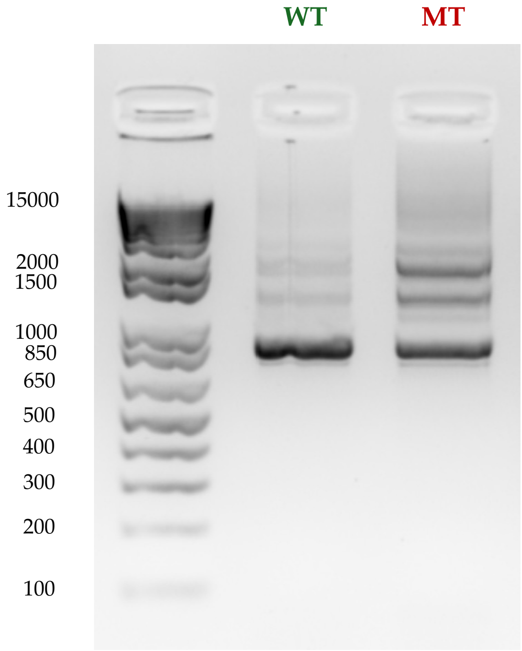

ATF6_minigene_ex1-2-13

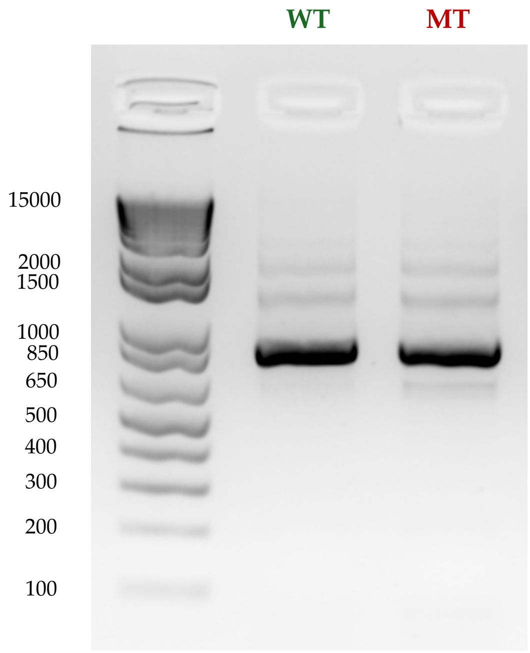

RHO_minigene_CACNA1F_int14-18

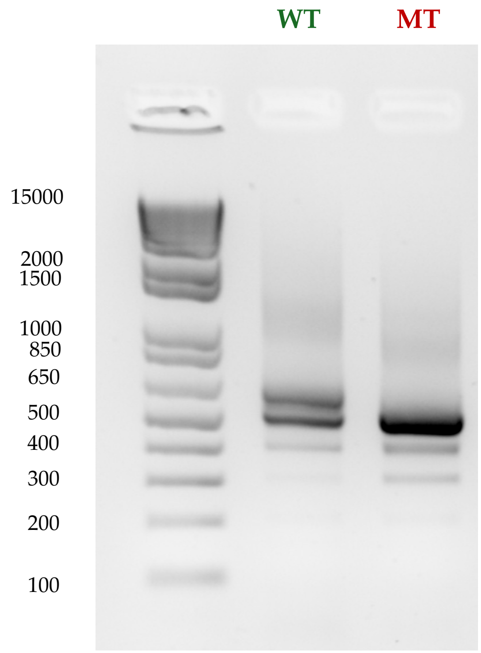

RHO_minigene_CHM_int9-11

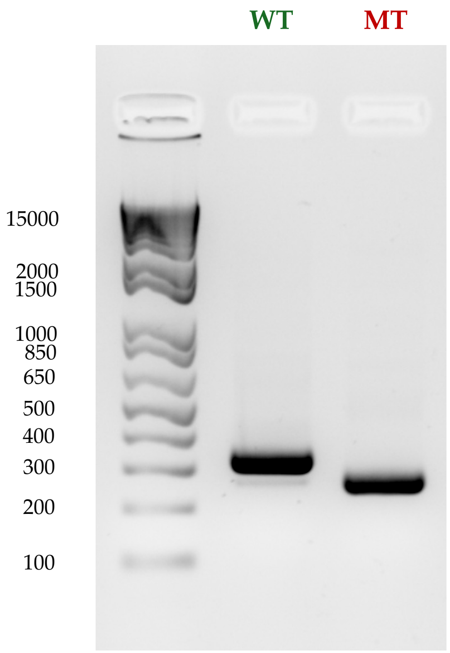

FZD4_minigene_ex1-2

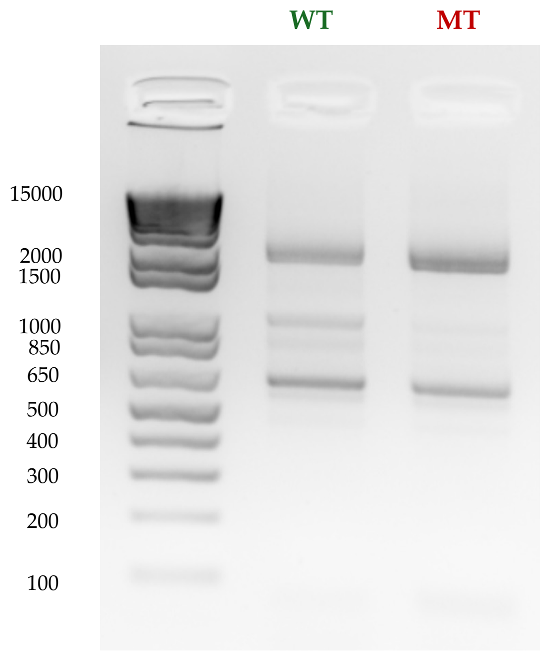

RHO_minigene_IMPG2_int15-18

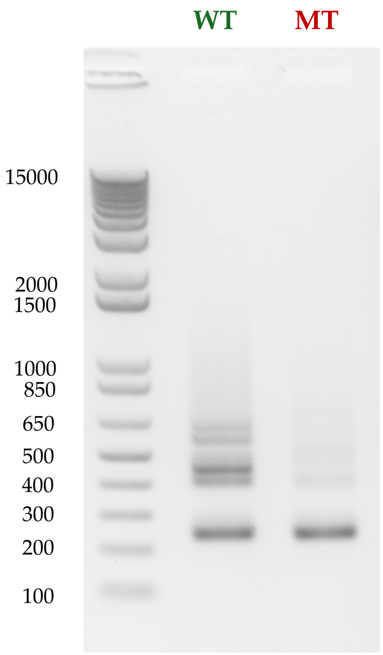

RHO_minigene_IMPG2_int16-17

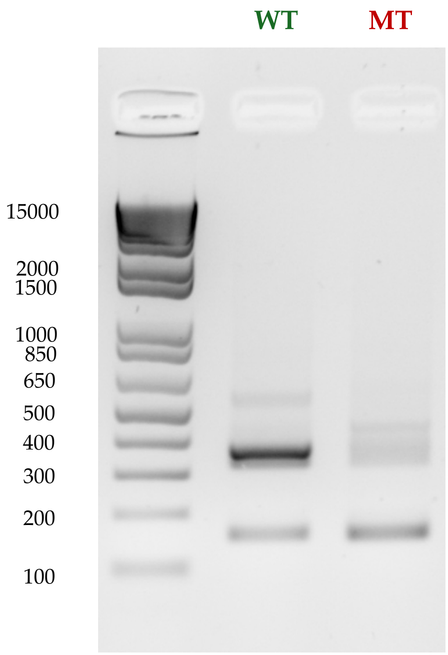

RHO_minigene_OCA2_int5-7

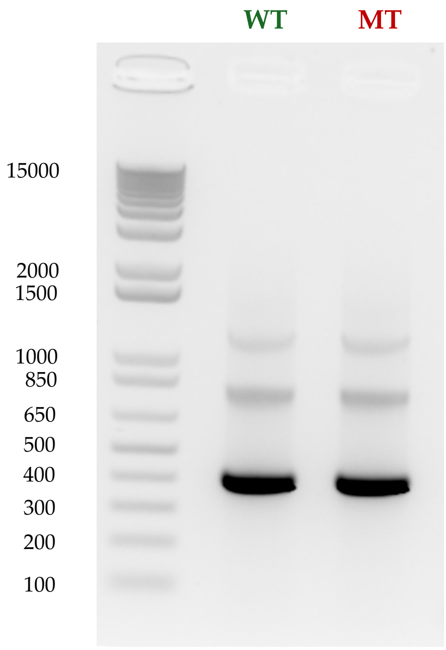

RHO_minigene_PDE6C_int3-4

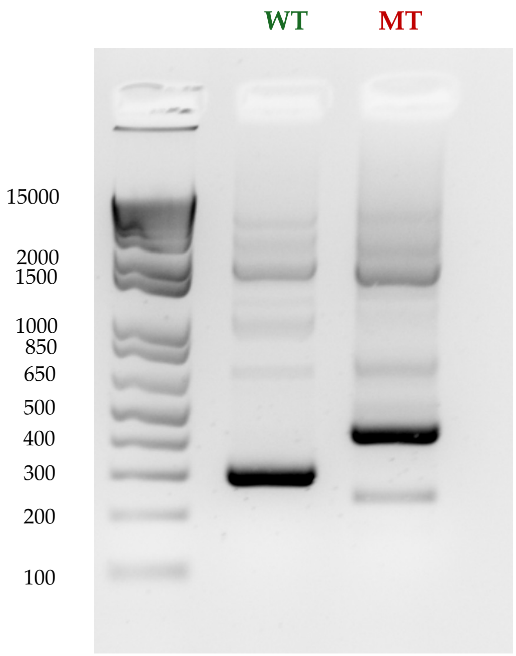

RHO_minigene_POC1B_int6-7

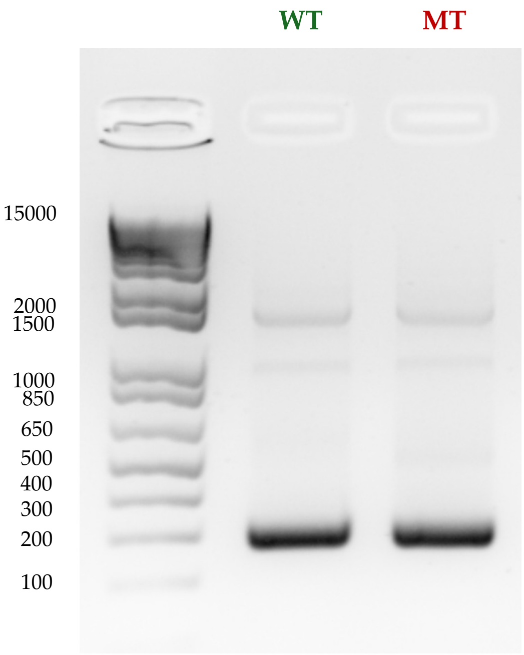

RHO_minigene_POC1B_int9-10

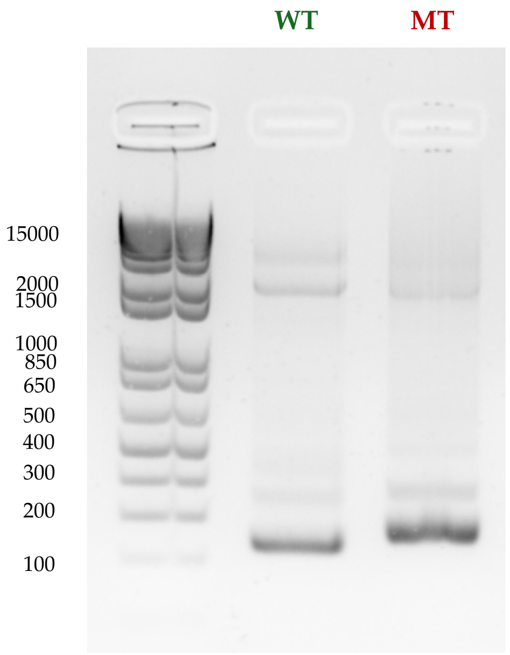

RHO_minigene_PROM1_int20-23

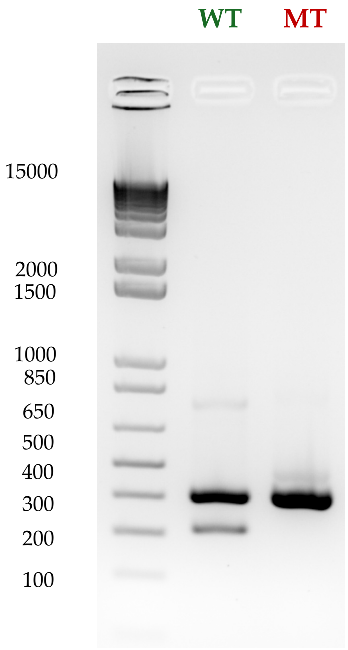

RHO_minigene_PROM1_int23-26

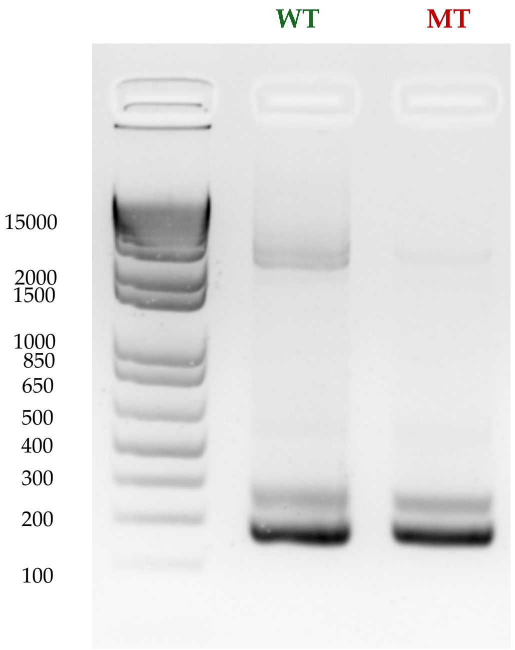

RHO_minigene_REEP6_int1-5

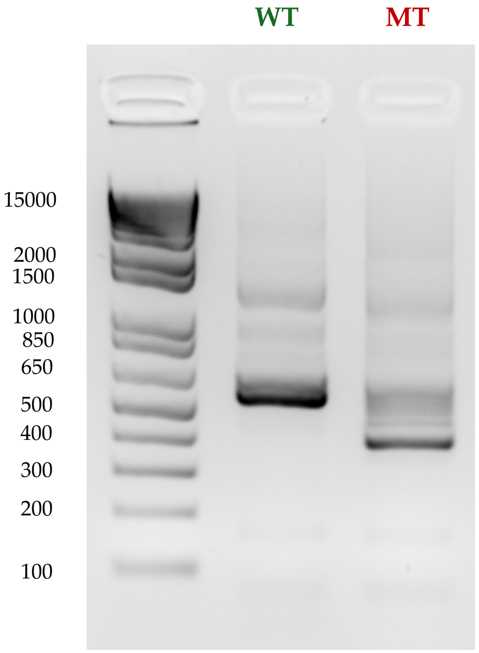

RHO_minigene_RPGR_int10-13

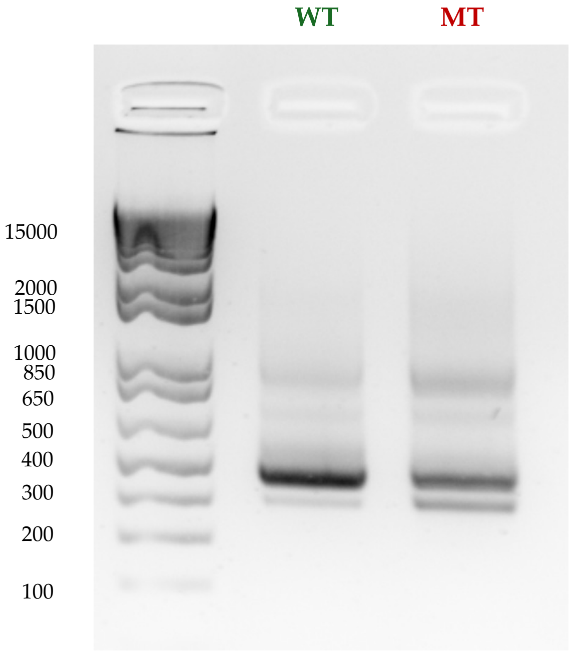

RHO_minigene_TIMP3_int1-3

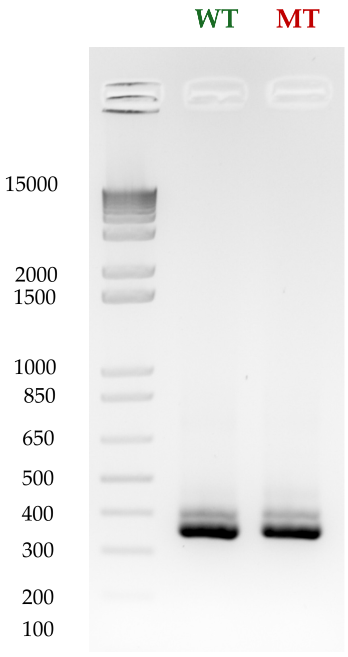

RHO_minigene_USH2A_int3

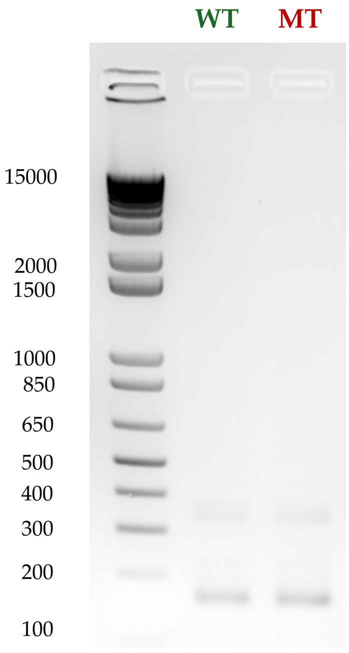

Supplement: Supplementary file 1 [file ijms-25-09569-s001.zip › Supplementary_materials/Manuscript_Figure_S3.pdf]
