# Supplementary material for: Nanopore Deep Sequencing as a Tool to Characterize and Quantify Aberrant Splicing Caused by Variants in Inherited Retinal Dystrophy Genes
Source: Int J Mol Sci. 2024 Sep 3;25(17):9569. doi: 10.3390/ijms25179569 (PMC11395040; doi:10.3390/ijms25179569)
Supplement: Supplementary file 1 [file ijms-25-09569-s001.zip › Supplementary_materials/Manuscript_Table_S1-3.pdf]

Variant (cNomen)	Distance NAS	Distance NDS	Predicted effect NAS	Predicted effect NDS	Effect score NAS (%)	Effect score NDS (%)	SpliceAI AL	SpliceAI DL	Pangolin SL
NM_000350.2:c.573C>T	-2	195	Weaker	No effect	-1,10	0	0	0	0
NM_000350.2:c.5586T>A	-1	128	Weaker	No effect	-16,70	0	0,01	0	0,03
NM_007348.3:c.1096-15G>A	15	106	Weaker	No effect	-7,11	0	0,17	0,25	0,44
NM_007348.3:c.1534-9A>G	9	79	Weaker	No effect	-41,61	0	0,76	0,05	0,81
NM_005183.4:c.2239+5C>G	-125	-5	No effect	Stronger	0	4,33	0	0,23	0,47
NM_000390.4:c.1413G>C	-63	0	Stronger	Weaker	1,00	-31,62	0,73	0,82	0,77
NM_012193.4:c.313A>G	-27	NA	Stronger	NA	0,02	NA	0	0	0
NM_016247.4:c.3423-7_3423-4del	4	214	Weaker	No effect	-19,67	0	0,38	0	0,33
NM_004523.3:c.1875+2T>A	-174	-2	No effect	No effect	0,00	0,00	0,11	0,91	0,73
NM_000275.3:c.574-53C>G	53	125	Weaker	No effect	-0,01	0	0	0	0
NM_006204.3:c.864+1G>A	-141	-1	No effect	Abolished	0	-8,06	0	0,99	0,8
NM_172240.2:c.677-2A>G	2	135	Abolished	No effect	-27,50	0	1	0,01	0,85
NM_172240.2:c.1033-327T>A	7	29	Abolished	No effect	-6,11	0	0,42	0	0,35
NM_006017.3:c.2358C>T	-77	15	Weaker	Weaker	-0,33	-0,63	0,22	0,29	0,32
NM_006017.3:c.2490-2A>G	2	25	Abolished	No effect	-66,94	0	0,51	0,41	0,42
NM_001329556.3:c.517G>A	-168	0	No effect	Weaker	0	-52,11	0	0,49	0,71
NM_001034853.1:c.1415-9A>G	9	100	Weaker	No effect	-36,00	0	0,95	0	0,8
NM_000362.4:c.205-3117T>C	3117	3228	No effect	No effect	0,00	0	0	0	0
NM_206933.2:c.652-22287T>C	22287	22419	No effect	No effect	0,00	0	0	0	0

Table S1: Splicing prediction scores for natural (canonical) splice sites closest to the variants. The effect score has been calculated from the average effect (transformed in percentages) predicted by SSF, MaxEnt, NNSPLICE, GeneSplicer. The distance is meant as basepairs between the variant and the splice site. Abbreviations: cNomen, Human Genome Variation Society (HGVS) cDNA-level nucleotide change nomenclature; NAS, natural acceptor splice site; NDS, natural donor splice site; AL, acceptor loss; DL, donor loss; SL, splice loss; NA, not applicable.

Variant (cNomen)	Distance CAS	Distance CDS	Predicted effect CAS	Predicted effect CDS	Effect score CAS (%)	Effect score CDS (%)	SpliceAI AG	SpliceAI DG	Pangolin SG
NM_000350.2:c.573C>T	-35	NA	Weaker	NA	-0,54	NA	0	0	0
NM_000350.2:c.5586T>A	-56	NA	NA	NA	NA	NA	0,07	0	0,04
NM_007348.3:c.1096-15G>A	2	NA	Created	NA	25,00	NA	0,22	0	0,18
NM_007348.3:c.1534-9A>G	1	NA	Created	NA	64,53	NA	0,89	0	0,82
NM_005183.4:c.2239+5C>G	NA	-45	NA	NA	NA	NA	0	0,11	0,21
NM_000390.4:c.1413G>C	NA	NA	NA	NA	NA	NA	0	0	0,01
NM_012193.4:c.313A>G	-1	-174	Created	NA	42,03	NA	0,72	0,01	0,68
NM_016247.4:c.3423-7_3423-4del	-77	NA	Stronger	NA	1,12	NA	0,57	0	0,21
NM_004523.3:c.1875+2T>A	70	-92	Stronger	NA	0,25	NA	0	0,06	0,03
NM_000275.3:c.574-53C>G	1	NA	Created	NA	4,27	NA	0	0	0
NM_006204.3:c.864+1G>A	NA	127	NA	NA	NA	NA	0	0,09	0,15
NM_172240.2:c.677-2A>G	-9	NA	Created	NA	8,94	NA	0,96	0	0,79
NM_172240.2:c.1033-327T>A	-2	-29	Created	NA	50,6	NA	0,6	0,41	0,58
NM_006017.3:c.2358C>T	3	NA	Stronger	NA	0,12	NA	0	0	0
NM_006017.3:c.2490-2A>G	259	NA	NA	NA	NA	NA	0,03	0	0,01
NM_001329556.3:c.517G>A	15	4/43	Weaker	Stronger	-16,64	1,23	0	0,46	0,2
NM_001034853.1:c.1415-9A>G	-1	NA	Created	NA	62,92	NA	0,98	0	0,87
NM_000362.4:c.205-3117T>C	12	NA	Weaker	NA	-19,90	NA	0	0	0,02
NM_206933.2:c.652-22287T>C	29	NA	Stronger	NA	6,98	NA	0	0	0,01

Table S2: Splicing prediction scores for cryptic splice sites created or affected by the variants. The effect score has been calculated from the average effect (transformed in percentages) predicted by SSF, MaxEnt, NNSPLICE, GeneSplicer. The distance is meant as basepairs between the variant and the splice site. Abbreviations: cNomen, Human Genome Variation Society (HGVS) cDNA-level nucleotide change nomenclature; CAS, cryptic acceptor splice site; CDS, cryptic donor splice site; AG, acceptor gain; DG, donor gain; SG, splice gain; NA, not applicable.

Variant (cNomen)	Distance AS	Distance DS	ESS WT	ESS MT	ESE WT	ESE MT	ESS/ESE ratio WT	ESS/ESE ratio MT	EX-skip predicted effect
NM_000350.2:c.573C>T	-2	195	3	5	29	29	0,1	0,17	MT higher change of exon skipping
NM_000350.2:c.5586T>A	-1	128	7	7	24	24	0,29	0,29	Comparable change of exon skipping
NM_007348.3:c.1096-15G>A	2	106	38	50	61	70	0,62	0,71	MT higher change of exon skipping
NM_007348.3:c.1534-9A>G	1	79	17	22	49	49	0,35	0,45	MT higher change of exon skipping
NM_005183.4:c.2239+5C>G	-125	-5	57	57	73	73	0,78	0,78	Comparable change of exon skipping
NM_000390.4:c.1413G>C	-63	0	4	4	39	38	0,1	0,11	MT higher change of exon skipping
NM_012193.4:c.313A>G	-27	NA	46	48	46	48	1	1	Comparable change of exon skipping
NM_016247.4:c.3423-7_3423-4del	-77	214	52	102	244	283	0,21	0,36	WT higher chance of exon skipping
NM_004523.3:c.1875+2T>A	-174	-26	68	61	117	96	0,58	0,64	MT higher change of exon skipping
NM_000275.3:c.574-53C>G	53	125	25	25	80	80	0,31	0,31	Comparable change of exon skipping
NM_006204.3:c.864+1G>A	-141	-1	4	4	53	53	0,08	0,08	Comparable change of exon skipping
NM_172240.2:c.677-2A>G	62	135	29	12	84	68	0,35	0,18	WT higher chance of exon skipping
NM_172240.2:c.1033-327T>A	2	29	25	26	2	3	12,5	8,67	WT higher chance of exon skipping
NM_006017.3:c.2358C>T	-77	15	32	38	19	15	1,68	2,53	MT higher change of exon skipping
NM_006017.3:c.2490-2A>G	-259	25	3	279	27	76	0,11	3,67	MT higher change of exon skipping
NM_001329556.3:c.517G>A	-168	0	0	0	21	21	0	0	Comparable change of exon skipping
NM_001034853.1:c.1415-9A>G	1	100	32	35	117	125	0,27	0,28	MT higher change of exon skipping
NM_000362.4:c.205-3117T>C	-283	11	131	125	249	252	0,53	0,5	WT higher chance of exon skipping
NM_206933.2:c.652-22287T>C	-29	169	162	162	123	140	1,32	1,16	WT higher chance of exon skipping

Table S3: Exonic splicing enhancer and silencer sequences predictions for exon or pseudoexon closest to the variants. Abbreviations: cNomen, Human Genome Variation Society (HGVS) cDNA-level nucleotide change nomenclature; AS, acceptor splice site; DS, donor splice site; WT, reference sequence; MT, mutant (variant) sequence; ESE, exonic splicing enhancer sequence; ESS, exonic splicing silencer sequence; NA, not applicable.
