# Supplementary material for: Nanopore Deep Sequencing as a Tool to Characterize and Quantify Aberrant Splicing Caused by Variants in Inherited Retinal Dystrophy Genes
Source: Int J Mol Sci. 2024 Sep 3;25(17):9569. doi: 10.3390/ijms25179569 (PMC11395040; doi:10.3390/ijms25179569)
Supplement: Supplementary file 1 [file ijms-25-09569-s001.zip › Supplementary_materials/Manuscript_Table_S4.pdf]

Table S4: Splicing strength scores from Alamut Visual Plus for natural (canonical) splice sites of all native exons included in the minigene constructs. Strength scores were transformed into percentages. Abbreviations: ex, exon.

	Acceptor					Donor				
**Exon**	**SSF**	**MaxEnt**	**NNSPLICE**	**GeneSplicer**	**Average**	**SSF**	**MaxEnt**	**NNSPLICE**	**GeneSplicer**	**Average**
**RHO ex3**						81.14	78.33	96.00	44.50	**74.99**
**RHO ex5**	86.29	46.00	94.00	42.10	**67.10**					
**ABCA4 ex5**	84.38	61.25	94.00	32.38	**68.00**	84.90	78.83	93.00	0.00	**64.18**
**ABCA4 ex6**	86.80	66.88	99.00	82.33	**83.75**	83.42	69.75	95.00	16.75	**66.23**
**ABCA4 ex39**	89.32	62.69	89.00	49.86	**72.72**	72.92	35.83	0.00	14.42	**30.79**
**ABCA4 ex40**	81.53	26.25	48.00	25.14	**45.23**	80.40	82.42	100.00	33.54	**74.09**
**ABCA4 ex41**	90.76	62.81	97.00	46.43	**74.25**	94.67	87.58	100.00	19.38	**75.41**
**ATF6 ex1**						83.62	66.08	97.00	13.83	**65.13**
**ATF6 ex2**	76.47	55.50	94.00	49.38	**68.84**	83.26	55.75	97.00	6.13	**60.53**
**ATF6 ex9**	81.30	46.94	82.00	15.62	**56.46**	96.95	92.33	100.00	0.00	**72.32**
**ATF6 ex13**	93.63	58.00	93.00	40.81	**71.36**	94.67	89.75	100.00	18.50	**75.73**
**CACNA1F ex15**	87.91	60.13	97.00	40.81	**71.46**	84.59	81.58	95.00	46.58	**76.94**
**CACNA1F ex16**	78.78	48.38	60.00	39.81	**56.74**	0.00	0.00	0.00	0.00	**0.00**
**CACNA1F ex17**	0.00	0.00	57.00	6.95	**15.99**	81.35	63.50	0.00	16.29	**40.29**
**CACNA1F ex18**	87.94	52.31	91.00	41.29	**68.13**	87.13	84.17	93.00	29.54	**73.46**
**CHM ex10**	86.13	59.94	100.00	19.05	**66.28**	94.98	92.33	100.00	38.75	**81.52**
**CHM ex11**	91.45	52.63	88.00	18.05	**62.53**	87.85	73.00	98.00	8.17	**66.75**
**FZD4 ex1**						79.78	67.25	53.00	48.96	**62.25**
**FZD4 ex2**	89.51	56.25	91.00	37.33	**68.52**					
**IMPG2 ex16**	85.05	43.56	94.00	18.29	**60.22**	87.85	73.00	100.00	18.17	**69.75**
**IMPG2 ex17**	84.09	52.44	84.00	22.90	**60.86**	77.17	35.83	45.00	16.00	**43.50**
**IMPG2 ex18**	89.73	60.50	94.00	44.14	**72.09**	72.34	44.33	87.00	0.00	**50.92**
**OCA2 ex6**	85.70	52.25	81.00	20.67	**59.90**	0.00	0.00	0.00	0.00	**0.00**
**OCA2 ex7**	0.00	0.00	0.00	0.00	**0.00**	72.71	19.75	0.00	0.00	**23.12**
**PDE6C ex4**	80.85	56.81	84.00	35.57	**64.31**	0.00	32.25	0.00	0.00	**8.06**
**PROM1 ex21**	88.38	59.00	65.00	23.05	**58.86**	86.80	72.50	99.00	9.75	**67.01**
**PROM1 ex22**	99.06	75.88	99.00	57.57	**82.88**	74.34	66.33	90.00	7.88	**59.64**
**PROM1 ex23**	72.69	64.13	90.00	48.86	**68.92**	82.03	73.75	98.00	7.25	**65.26**

**PROM1 ex24**	88.60	52.50	99.00	22.43	**65.63**	74.78	73.50	97.00	0.00	**61.32**
**PROM1 ex25**	85.76	62.63	98.00	21.38	**66.94**	81.78	60.92	98.00	0.00	**60.17**
**PROM1 ex26**	90.45	48.63	83.00	0.00	**55.52**	82.52	75.50	97.00	0.00	**63.76**
**REEP6 ex2**	85.09	55.00	54.00	32.57	**56.67**	73.82	70.08	83.00	36.00	**65.73**
**REEP6 ex3**	79.32	71.94	96.00	46.76	**73.50**	79.47	66.08	41.00	19.13	**51.42**
**REEP6 ex4**	73.69	55.44	0.00	40.43	**42.39**	76.34	68.92	70.00	0.00	**53.81**
**REEP6 ex5**	0.00	3.13	0.00	0.00	**0.78**	0.00	74.83	50.00	20.50	**36.33**
**RPGR ex11**	85.60	47.81	0.00	0.00	**33.35**	81.98	74.00	93.00	0.00	**62.25**
**RPGR ex12**	81.92	50.06	75.00	27.71	**58.67**	73.77	45.50	91.00	0.00	**52.57**
**RPGR ex13**	94.80	52.31	96.00	36.38	**69.87**	72.04	59.00	88.00	1.96	**55.25**
**TIMP3 ex2**	88.06	64.06	96.00	59.14	**76.82**	89.61	85.75	99.00	44.13	**79.62**
