# Supplementary material for: Nanopore Deep Sequencing as a Tool to Characterize and Quantify Aberrant Splicing Caused by Variants in Inherited Retinal Dystrophy Genes
Source: Int J Mol Sci. 2024 Sep 3;25(17):9569. doi: 10.3390/ijms25179569 (PMC11395040; doi:10.3390/ijms25179569)
Supplement: Supplementary file 1 [file ijms-25-09569-s001.zip › Supplementary_materials/Manuscript_Table_S5.pdf]

**Table S1.** List of loci of interest for eye disorders

| List | Loci |
| --- | --- |
| Eye disorders | *AAAS, AASS, ABCA1, ABCA4, ABCB6, ABCC6, ABCD1, ABHD12, ACBD5, ACO2, ACTA2, ACTB, ACTG1, ADAM9, ADAMTS10, ADAMTS17, ADAMTS18, ADAMTSL1, ADAMTSL4, ADGRA3, ADGRV1, ADIPOR1, ADORA2A, AGBL1, AGBL5, AGK, AGPS, AGXT, AHI1, AHR, AIPL1, AKR1E2, ALDH18A1, ALDH1A3, ALDH3A2, ALG3, ALMS1, ALPK1, AMACR, ANAPC1, ANXA8, AP3B1, AP3B2, APC, ARHGEF16, ARHGEF17, ARHGEF18, ARL13B, ARL2, ARL2BP, ARL3, ARL6, ARMC9, ARMS2, ARSG, ARSL, ASPH, ASRGL1, ATF6, ATOH7, ATXN7, B3GALNT2, B3GALT6, B3GLCT, B4GALT7, BAP1, BBIP1, BBS1, BBS10, BBS12, BBS2, BBS4, BBS5, BBS7, BBS9, BCOR, BEST1, BFSP1, BFSP2, BLOC1S3, BLOC1S6, BMP4, BPHL, BUB1B, C12orf57, C19orf12, C1QTNF5, C2, C3, CA4, CABP4, CACNA1F, CACNA2D4, CANT1, CAPN5, CBS, CC2D2A, CCDC28B, CCDC39, CCDC40, CCDC66, CCT2, CDH16, CDH23, CDH3, CDHR1, CDK10, CEP104, CEP120, CEP164, CEP250, CEP290, CEP41, CEP78, CERKL, CFAP410, C21orf2, CFAP418, CFB, CFH, CHD7, CHM, CHMP4B, CHN1, CHRDL1, CHST14, CHST6, CIB2, CISD2, CLCC1, CLCN2, CLDN19, CLN3, CLN5, CLN6, CLN8, CLPB, CLRN1, CLUAP1, CNBP, CNGA1, CNGA3, CNGB1, CNGB3, CNNM4, COA8, COG5, COL11A1, COL17A1, COL18A1, COL2A1, COL4A1, COL4A3, COL4A4, COL4A5, COL5A1, COL7A1, COL8A2, COL9A1, COL9A2, COL9A3, CPAMD8, CPLANE1, CRB1, CRB2, CREBBP, CRIM1, CRX, CRYAA, CRYAB, CRYBA1, CRYBA2, CRYBA4, CRYBB1, CRYBB2, CRYBB3, CRYGA, CRYGB, CRYGC, CRYGD, CRYGS, CSPP1, CTC1, CTDP1, CTNNA1, CTNNB1, CTNS, CTSD, CWC27, CYP1B1, CYP27A1, CYP4V2, CYP51A1, DAG1, DCN, DDX58, DHCR7, DHDDS, DHX32, DHX38, DKC1, DMPK, DNAAF1, DNAAF2, DNAH11, DNAH5, DNAH9, DNAI1, DNAI2, DNAJC17, DNAL1, DNM1L, DPYD, DRAM2, DSCAML1, DST, DTHD1, DTNBP1, DYNC2H1, DYNC2I2, EBP, EFEMP1, EIF2B2, ELOVL1, ELOVL4, ELP1, ELP4, EMC1, EP300, EPG5, EPHA2, ERBB3, ERCC1, ERCC2, ERCC3, ERCC5, ERCC6, ERCC8, ESCO2, ESPN, ETFDH, EXOSC2, EYA1, EYS, FA2H, FAM111A, FAM126A, FAM161A, FAM57B, FAR1, FBLN5, FBN1, FBN2, FDFT1, FDXR, FGF10, FGFR2, FGFR3, FKRP, FKTN, FLNB, FLVCR1, FNBP4, FOXC1, FOXC2, FOXD3, FOXE3, FOXI2, FOXL2, FRAS1, FREM1, FREM2, FRMD7, FSCN2, FTL, FYCO1, FZD4, FZD5, GALK1, GALT, GCNT2, GDF3, GDF6, GEMIN4, GFER, GGCX, GJA1, GJA3, GJA8, GJB6, GLA, GMPPA, GNAQ, GNAS, GNAT1, GNAT2, GNB3, GNPAT, GNPTG, GPATCH3, GPKOW, GPR143, GPR179, GPR45, GRHL2, GRID2, GRIP1, GRK1, GRM6, GRN, GSN, GUCA1A, GUCA1B, GUCY2D, GZF1, HADHA, HARS1, HCCS, HESX1, HGSNAT, HK1, HKDC1, HMCN1, HMGB3, HMX1, HOXA1, HPS1, HPS3, HPS4, HPS5, HPS6, HSF4, HSPG2, HTRA1, IARS2, IDH3A, IDH3B, IFIH1, IFT122, IFT140, IFT172, IFT27, IFT43, IFT52, IFT88, IGBP1, IMPDH1, IMPG1, IMPG2, INPP5E, INPP5K, INTS1, INVS, IPO13, IQCB1, IRX1, IRX5, ITM2B, ITPR1, JAG1, JAM3, KAT6B, KATNIP, KCNA4, KCNJ13, KCTD7, KCNV2, KERA, KIAA0586, KIAA1549, KIF11, KIF21A, KIF3B, KIF7, KIT, KIZ, KLHL7, KRT12, KRT3, LAMA1, LAMB2, LARGE1, LCA5, LCAT, LEMD2, LIG3, LIM2, LINC01476, LMX1B, LONP1, LOXHD1, LOXL3, LRAT, LRIT3, LRMDA, LRP2, LRP5, LRPAP1, LRRTM4, LSS, LTBP2, LYST, LZTFL1, MAB21L1, MAB21L2, MAF, MAFB, MAK, MAN2B1, MAP3K1, MAP7D2, MAPKAPK3, MAPRE2, MARK3, MCOLN1, MED12, MERTK, MFN2, MFRP, MFSD6L, MFSD8, MIEF1, MIP, MIR184, MIR204, MITF, MKKS, MKS1, MMACHC, MOCS1, MOCS2, MPDZ, MPRIP, MRTFA, MRTFB, MT-ATP6, MT-ND1, MT-ND4, MT-ND5, MT-ND6, MTR, MTRFR, MTTP, MVK, MYF5, MYH9, MYO7A, MYOC, MYRF, NAA10, NAALADL1, NACC1, NBAS, NDP, NDRG4, NDUFS1, NDUFS2, NEK1, NEK2, NEU1, NEUROD1, NEUROG2, NF2, NFE2L1, NGLY1, NHS, NINJ1, NLRP1, NME8, NMNAT1, NOD2, NOTCH2, NPHP1, NPHP3, NPHP4, NR2E3, NR2F1, NR4A3, NRF1, NRL, NUDT19, NUMB, NYX, OAT, OCA2, OCRL, OFD1, OPA1, OPA3, OPN1LW, OPN1MW, OPN1SW, OPTN, OR2M7, OR2W3, OTOGL, OTX2, OVOL2, P3H2, P4HA2, PANK2, PANK4, PARVA, PAX2, PAX6, PCARE, C2orf71, PCDH15, PCYT1A, PDCD2, PDE6A, PDE6B, PDE6C, PDE6D, PDE6G, PDE6H, PDZD7, PET100, PEX1, PEX10, PEX11B, PEX12, PEX13, PEX14, PEX16, PEX19, PEX2, PEX26, PEX3, PEX5, PEX6, PEX7, PGK1, PHOX2A, PHYH, PIBF1, PIGA, PIGL, PIGN, PIK3R1, PIKFYVE, PITPNM3, PITX2, PITX3, PKM, PLA2G5, PLCB3, PLD4, PLK4, PLOD1, PLXND1, PMM2, PNPLA6, POC1B, POC5, PODNL1, POLG, POLR2D, POMGNT1, POMT1, POMT2, POMZP3, PORCN, PPP1R21, PPT1, PQBP1, PRCD, PRDM13, PRDM5, PRKCG, PRKRA, PROM1, PROS1, PRPF3, PRPF31, PRPF4, PRPF6, PRPF8, PRPH2, PRPS1, PRR12, PRSS56, PRTFDC1, PTCH1, PTEN, PUF60, PXDN, RAB18, RAB28, RAB3GAP1, RAB3GAP2, RARB, RAX, RAX2, RB1, RBP3, RBP4, RBPJ, RCBTB1, RD3, RDH11, RDH12, RDH5, RECQL4, REEP6, RELN, REV3L, RGL4, RGR, RGS9, RGS9BP, RHEX, RHO, RHOD, RIC1, RIMS1, RIMS2, RLBP1, RNF7, RNLS, ROBO3, ROM1, RP1, RP1L1, RP2, RP9, RPE65, RPGR, RPGRIP1, RPGRIP1L, RPIA, RRAGA, RS1, RSC1A1, RSPH4A, RSPH9, RTN4IP1, SAG, SALL4, SAMD11, SBF2, SC5D, SCAPER, SCLT1, SCO2, SDCCAG8, SEC23A, SEMA4A, SEMA6B, SF3B2, SGPP2, SH3PXD2B, SHH, SIGMAR1, SIL1, SIPA1L3, SIX3, SIX6, SLC16A12, SLC24A1, SLC24A5, SLC25A46, SLC2A1, SLC33A1, SLC37A3, SLC38A8, SLC39A5, SLC39A12, SLC45A2, SLC4A11, SLC4A4, SLC4A7, SLC52A2, SLC66A1, SLC7A14, SLC9A8, SLITRK6, SMCHD1, SMOC1, SNRNP200, SOX10, SOX2, SPAST, SPATA7, SPG7, SPINT2, SPP2, SRD5A3, SRF, STK19, STK19B, STN1, STRA6, STRC, STS, SUOX, SYTL4, TACSTD2, TAF1A, TAPT1, TBC1D20, TBC1D32, TBX2, TBX22, TCF20, TCF4, TCTN1, TCTN2, TDRD7, TEAD1, TECPR2, TEK, TENM3, TET2, TET3, TFAP2A, TGFB1, TGFBI, TIMM8A, TIMP3, TINF2, TJP1, TLR3, TLR4, TMEM114, TMEM126A, TMEM138, TMEM216, TMEM218, TMEM231, TMEM237, TMEM67, TMEM70, TMEM98, TOP2B, TOPORS, TP63, TPP1, TRAF3IP1, TRAPPC11, TREX1, TRIM32, TRIM44, TRNT1, TRPM1, TSC1, TSC2, TSPAN11, TSPAN12, TTC21B, TTC8, TTLL10, TTLL5, TTPA, TUB, TUBB2B, TUBB3, TUBGCP4, TUBGCP6, TULP1, TYR, TYRP1, UBAP1L, UBE3B, UBIAD1, UNC119, UNC45B, UNC80, USH1C, USH1G, USH2A, USP16, USP21, USP45, VAX1, VAX2, VCAN, VHL, VIM, VLDLR, VPS13B, VSX1, VSX2, WASF3, WDPCP, WDR19, WDR87, WFS1, WHRN, WNT3, WRN, WT1, XPNPEP2, XYLT2, YAP1, ZEB1, ZEB2, ZNF408, ZNF423, ZNF469, ZNF513* |
