# Supplementary material for: Nanopore Deep Sequencing as a Tool to Characterize and Quantify Aberrant Splicing Caused by Variants in Inherited Retinal Dystrophy Genes
Source: Int J Mol Sci. 2024 Sep 3;25(17):9569. doi: 10.3390/ijms25179569 (PMC11395040; doi:10.3390/ijms25179569)
Supplement: Supplementary file 1 [file ijms-25-09569-s001.zip › Supplementary_materials/Manuscript_Table_S6-7.pdf]

**Table S6: Primers for minigene inserts.** Abbreviations: F, forward; R, reverse.

| Minigene | F primer | R primer | F primer | R primer | F primer | R primer |
|---|---|---|---|---|---|---|
| **RHO_minigene_ABCA4_int4-6** | AGCCTCATGAACTATAGCACTAGC | TGAGACCATTTCCAGAGGAAGAAG | | | | |
| **RHO_minigene_ABCA4_int38-41** | CCCACGAAGTTTGCAACGATATAA | AAACTACCAGCACTAGGAGGTTAC | | | | |
| **RHO_minigene_ATF6_int8-9** | TTTTCTCTCCCTGCTGGAAAAG | TCCCAGCTGGATCATGAG | | | | |
| **ATF6_minigene_ex1-2-9** | GTATTTTGTCCGCCTGCCGCCGCC GTC | AGAATTCTGAAAACCTCCTAAAGAC GTCCAGTG | TAGGAGGTTTTCAGAATT CTTGTTAGTAATGTTCC | GAATTCAATAGCCAATTC CCTTATGTAGC | GGGAATTGGCTATTGAATT CTTTCCTCTTGGGTG | GGTTTAAACGGGCCCTCTAGGTA TCGAGTACCCATCAATACGAC |
| **ATF6_minigene_ex1-2-13** | GTATTTTGTCCGCCTGCCGCCGCC GTC | AGAATTCTGAAAACCTCCTAAAGAC GTCCAGTG | TAGGAGGTTTTCAGAATT CTTGTTAGTAATGTTCC | GTGTTACTGAGCCAATT CCCTTATGTAGC | GGGAATTGGCTCAGTAAC ACTTATATAGCTTGGTC | GGTTTAAACGGGCCCTCTAGGG CAAGAAAGCTATGGGTATTC |
| **RHO_minigene_CACNA1F_int14-18** | GCTTCCCCCACTTCTTACTCTC | TAACAAAAATTAGCCGAGTGTGGTG | | | | |
| **RHO_minigene_CHM_int9-11** | GTCCGTGCATAAGTGTTTTCC | TTCATTGCAGCGAAGATTTGG | | | | |
| **FZD4_minigene_ex1-2** | GCCCGGGACGTCTAAAATCCCACA | CTCACAGCTCAAATCCCACCCTG | | | | |
| **RHO_minigene_IMPG2_int15-18** | TGCCAGTCACTTTCCTTCCTAGGC | TCTGCCACTGGCTATCACTGTCATC | | | | |
| **RHO_minigene_IMPG2_int16-17** | GGAAAGGCATGGTTGAGTGTGAC | GCAGGAGTGTGCACTTTCAGAG | | | | |
| **RHO_minigene_OCA2_int5-7** | CCCAGAGAAAGGAAACCTATGCTA | CTTAGCAGATGTCCACAAACACAG | | | | |
| **RHO_minigene_PDE6C_int3-4** | ACCTATTAATTTGAGGAGCA | CTTTGGAAGACAGTCTGCC | | | | |
| **RHO_minigene_POC1B_int6-7** | CGAATGGACTCCTTTTGGAAGGACT | ACTCCCAAAGGCAGGAGAGTAG | | | | |
| **RHO_minigene_POC1B_int9-10** | ATGCCGTGTTTCCTCCATAGG | ACGGCAGTGCTGTTTTTC | | | | |
| **RHO_minigene_PROM1_int20-23** | TTTGCTTGTGTTGTAGTGCAAAGA | GGGTCTCAATCAATGTTTCACGTT | | | | |
| **RHO_minigene_PROM1_int23-26** | AACCTGCTGGCCATCCATGCTC | GACCGACCTCTGGCAAGCAA | | | | |
| **RHO_minigene_REEP6_int1-5** | TGAGATGGCTATCGTTGCAGATTA | GTTACGTACGGTCAGCTTCCAG | | | | |
| **RHO_minigene_RPGR_int10-13** | CCCCATCAGTGTTTTGTAGTTGTTG | CCTATGGCTTGACAGAAGGAAAAG | | | | |
| **RHO_minigene_TIMP3_int1-3** | CAGACTCTATTGCATCCCTTGCTA | CAAATTAGGAGATTTCCTCCCCCA | | | | |
| **RHO_minigene_USH2A_int3** | ATCAGCCTAAACCTAGCTTGGAAA | TGTCCTGATGTGGATGATGAAAGT | | | | |

**Table S7: Primers for Nanopore sequencing.** Abbreviations: F, forward; R, reverse.

| Region to be amplified | F primer | R primer |
|---|---|---|
| **RHO minigenes cDNA** | TTTCTGTTGGTGCTGATATTGCACTTGCCTGTCGCTCTATCTTC | ACTTGCCTGTCGCTCTATCTTCGCTTTTGTGACTCAACCAAGGA |
| **T7 BGH minigenes cDNA** | TTTCTGTTGGTGCTGATATTGCTAATACGACTCACTATAGG | ACTTGCCTGTCGCTCTATCTTCTAGAAGGCACAGTCGAGG |
| **CACNA1F ex15-17 blood cDNA** | TTTCTGTTGGTGCTGATATTGCCATCCTTGCTGCTTCTCCTCT | ACTTGCCTGTCGCTCTATCTTCCGCCCTTGTCCTTGGCAG |
| **KIF11 ex13-16 blood cDNA** | TTTCTGTTGGTGCTGATATTGCTTTTCTGCTATGGGCAGCTC | ACTTGCCTGTCGCTCTATCTTCCTTGGACACGGTAGCAGAGG |
