# Supplementary material for: Nanopore Deep Sequencing as a Tool to Characterize and Quantify Aberrant Splicing Caused by Variants in Inherited Retinal Dystrophy Genes
Source: Int J Mol Sci. 2024 Sep 3;25(17):9569. doi: 10.3390/ijms25179569 (PMC11395040; doi:10.3390/ijms25179569)
Supplement: Supplementary file 1 [file ijms-25-09569-s001.zip › Supplementary_materials/Manuscript_Table_S8.pdf]

Table S8: Demographic data of patients carrying the candidate splicing variants included in the study. Age at referral refers to the age of the index proband. Abbreviations: MD, macula dystrophy; RD, retinal dystrophy; ACHR, achromatopsia; CHM, choroideremia; EVR, exudative vitreoretinopathy; COD, cone dystrophy; CRD, cone-rod dystrophy; RP, retinitis pigmentosa; STGD, Stargardt's disease; VMD, vitelliform macular dystrophy; M, male; F, female; ND, no data.

Patient ID	Gene	Variant 1 (cNomen)	Variant 2 (cNomen)	Clinical phenotype	Sex	Age at referral	Family history	Previous report
**1**	*ABCA4*<br>*OCA2*	**NM_000350.2:c.573C>T**<br>**NM_000275.3:c.574-53C>G**	NM_000350.2:c.6479+1099G>C<br>NM_000275.2:c.1327G>A	MD/OCA	M	32	dominant	[1]
**2**	*ABCA4*	**NM_000350.2:c.5586T>A**	NM_000350.2:c.6124G>A	RD	M	56	dominant	[2]
**3**	*ATF6*	**NM_007348.3:c.1096-15G>A**	**NM_007348.3:c.1534-9A>G**	ACHR	F	0	negative	
**4**	*CACNA1F*	**NM_005183.4:c.2239+5C>G**		RD	M	28	X-linked	
**5**	*CHM*	**NM_000390.4:c.1413G>C**		CHM	M	44	X-linked	
**6**	*FZD4*	**NM_012193.4:c.313A>G**		EVR	M	4	dominant	
**7**	*IMPG2*	**NM_016247.4:c.3423-7_3423-4del**		MD	M	47	ND	[1,2]
**8**	*KIF11*	**NM_004523.3:c.1875+2T>A**		EVR	F	0	negative	[1]
**9**	*PDE6C*	**NM_006204.3:c.864+1G>A**	**NM_006204.3:c.864+1G>A**	COD	M	30	ND	
**10**	*POC1B*	**NM_172240.2:c.677-2A>G**	**NM_172240.2:c.1033-327T>A**	CRD	F	61	negative	
**11**	*PROM1*	**NM_006017.3:c.2358C>T**	**NM_006017.3:c.2358C>T**	STGD	M	49	negative	[1,2]
**12**	*PROM1*	**NM_006017.3:c.2490-2A>G**		STGD	M	6	negative	[2]
**13**	*REEP6*	**NM_001329556.3:c.517G>A**	**NM_001329556.3:c.517G>A**	RP	F	28	recessive	
**14**	*RPGR*	**NM_001034853.1:c.1415-9A>G**		RP	M	12	X-linked	[3]
**15**	*TIMP3*	**NM_000362.4:c.205-3117T>C**		VMD	F	32	negative	
**16**	*USH2A*	**NM_206933.2:c.652-22287T>C**	NM_206933.2:c.12066+4409C>G	RP	F	31	negative	

References

1. Maggi, J.; Koller, S.; Feil, S.; Bachmann-Gagescu, R.; Gerth-Kahlert, C.; Berger, W. Limited Added Diagnostic Value of Whole Genome Sequencing in Genetic Testing of Inherited Retinal Diseases in a Swiss Patient Cohort. International Journal of Molecular Sciences 2024, Vol. 25, Page 6540 2024, 25, 6540, doi:10.3390/IJMS25126540.
2. Maggi, J.; Koller, S.; Bähr, L.; Feil, S.; Pfiffner, F.K.; Hanson, J.V.M.; Maspoli, A.; Gerth-Kahlert, C.; Berger, W. Long-Range PCR-Based NGS Applications to Diagnose Mendelian Retinal Diseases. Int J Mol Sci 2021, 22, 1–34, doi:10.3390/ijms22041508.
3. Koller, S.; Beltraminelli, T.; Maggi, J.; Wlodarczyk, A.; Feil, S.; Baehr, L.; Gerth-Kahlert, C.; Menghini, M.; Berger, W. Functional Analysis of a Novel, Non-Canonical RPGR Splice Variant Causing X-Linked Retinitis Pigmentosa. Genes (Basel) 2023, 14, 934, doi:10.3390/GENES14040934/S1.
