# Supplementary material for: Nanopore Deep Sequencing as a Tool to Characterize and Quantify Aberrant Splicing Caused by Variants in Inherited Retinal Dystrophy Genes
Source: Int J Mol Sci. 2024 Sep 3;25(17):9569. doi: 10.3390/ijms25179569 (PMC11395040; doi:10.3390/ijms25179569)
Supplement: Supplementary file 1 [file ijms-25-09569-s001.zip › Supplementary_materials/Supplementary_Figure_S1.pdf]

Figure S1. Alamut Visual Plus splicing predictions. Screenshots from the splicing module of Alamut Visual Plus highlighting the differences in scores from SpliceSiteFinder-like, MaxEntScan, NNSPLICE, and GeneSplicer between the reference and the variant sequences for each variant included in the study. Acceptor splice site scores are shown in green; donor splice site scores are shown in blue. Unchanged score predictions are shown faded. Exon sequences are highlighted in light blue.

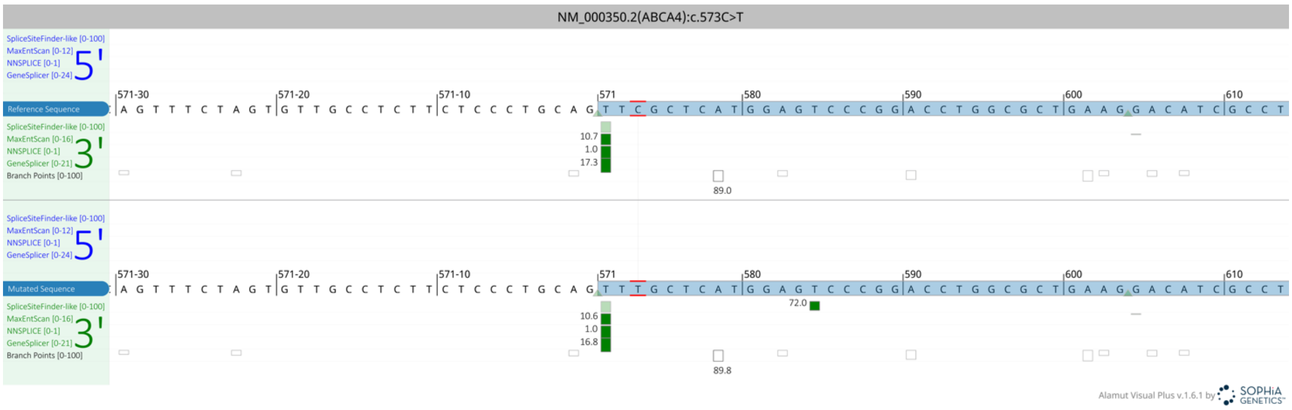

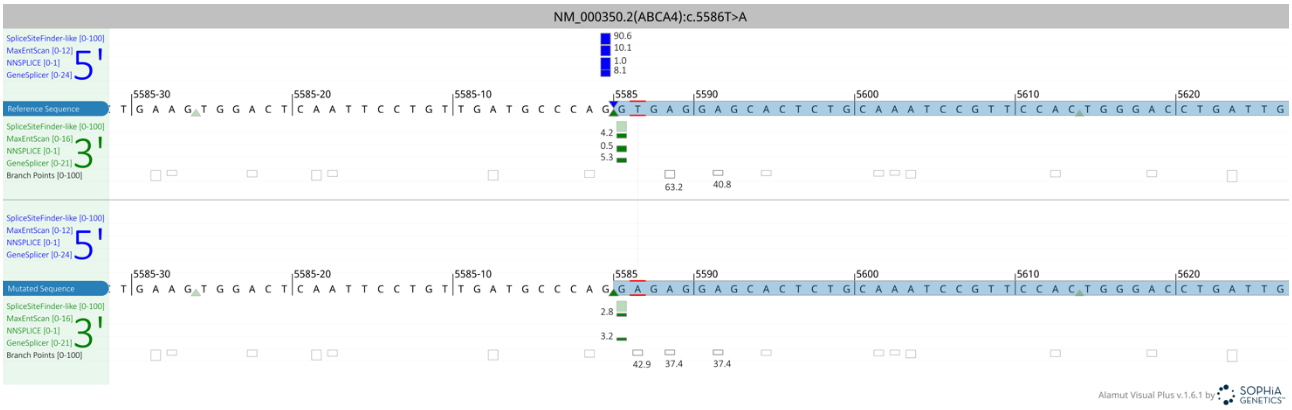

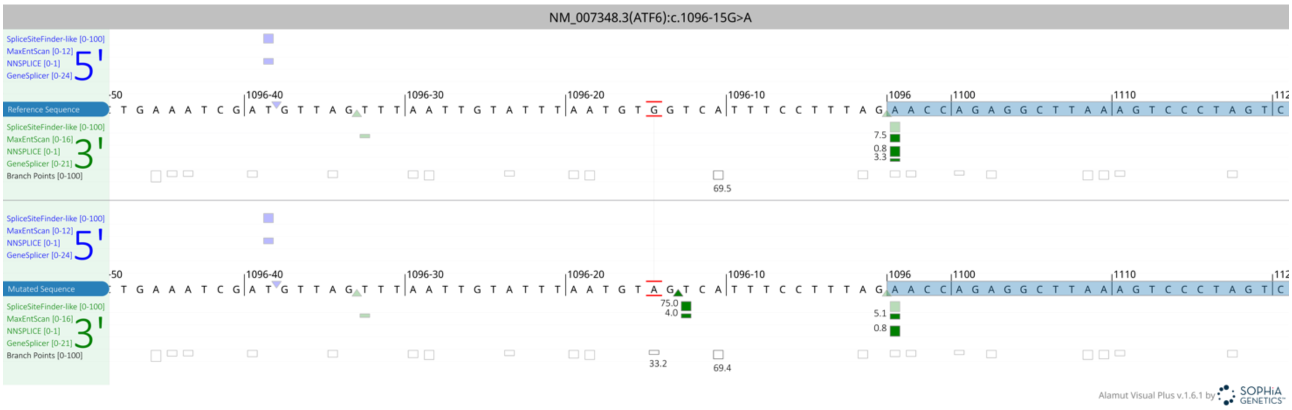

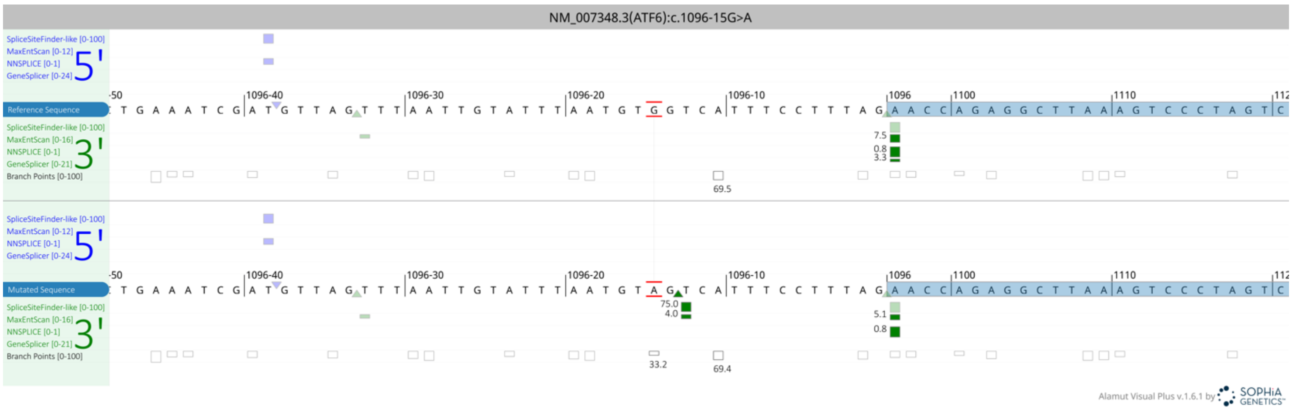
NM_007348.3(ATF6):c.1096-15G>A
SpliceSiteFinder-like [0-100]
MaxEntScan [0-12]
NNSPLICE [0-1]
GeneSplicer [0-24]
Reference Sequence
GeneSplicer [0-21]
Branch Points [0-100]
Mutated Sequence
Alamut Visual Plus v.1.6.1 by SOPHiA GENETICS

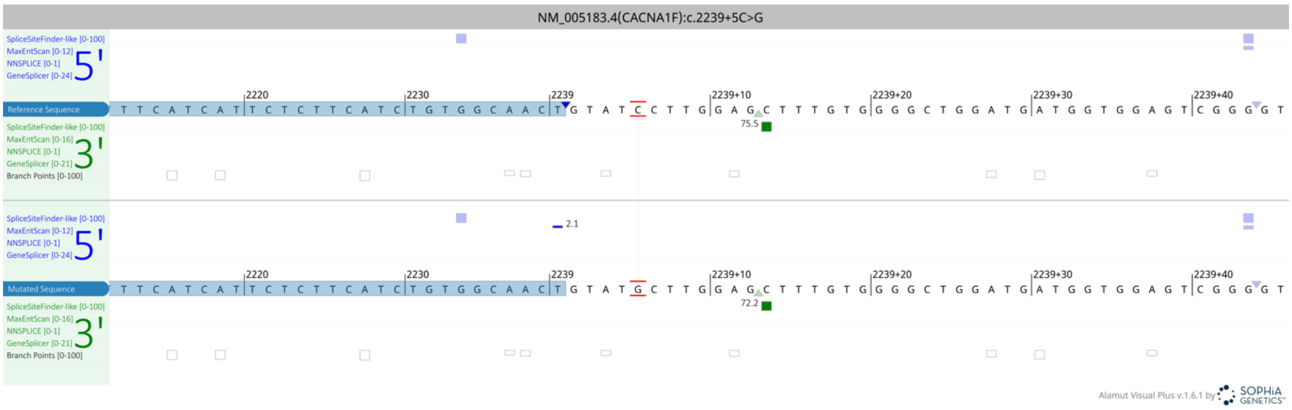
NM_005183.4(CACNA1F):c.2239+5C>G
SpliceSiteFinder-like [0-100]
MaxEntScan [0-12]
NNSPLICE [0-1]
GeneSplicer [0-24]
Reference Sequence
MaxEntScan [0-16]
GeneSplicer [0-21]
Branch Points [0-100]
Mutated Sequence
Alamut Visual Plus v.1.6.1 by SOPHiA GENETICS

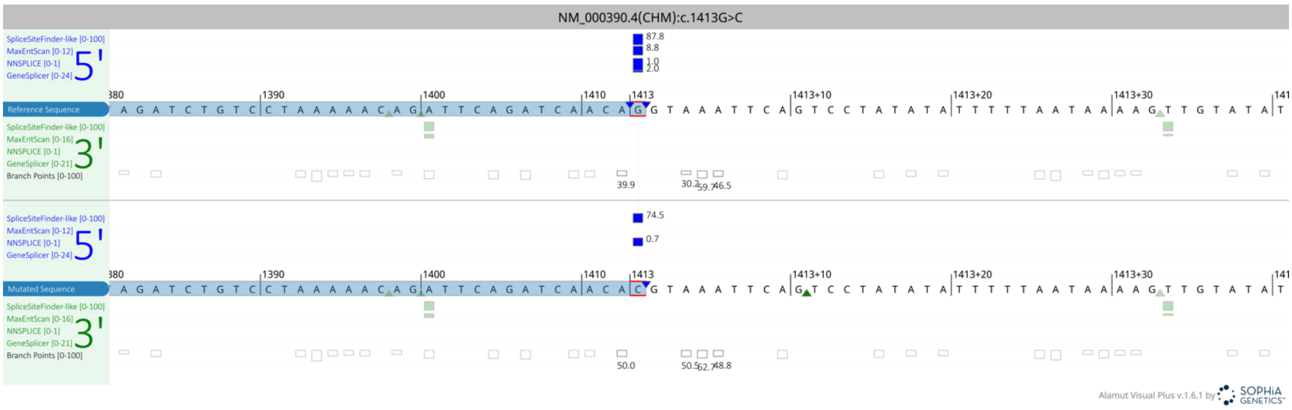
NM_000390.4(CHM):c.1413G>C
SpliceSiteFinder-like [0-100]
MaxEntScan [0-12]
NNSPLICE [0-1]
GeneSplicer [0-24]
Reference Sequence
MaxEntScan [0-16]
GeneSplicer [0-21]
Branch Points [0-100]
Mutated Sequence
Alamut Visual Plus v.1.6.1 by SOPHiA GENETICS

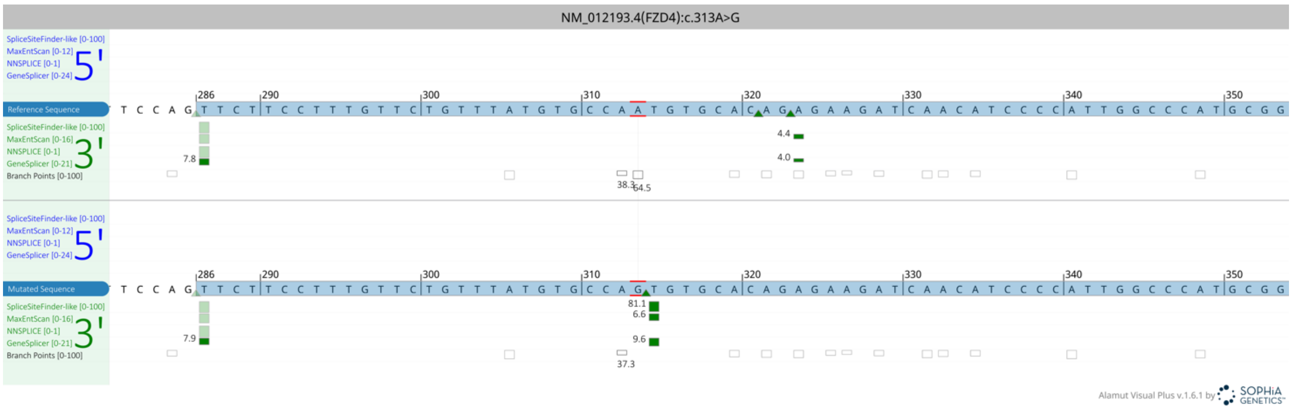
NM_012193.4(FZD4):c.313A>G
SpliceSiteFinder-like [0-100]
MaxEntScan [0-12]
NNSPLICE [0-1]
GeneSplicer [0-24]
Reference Sequence
MaxEntScan [0-16]
GeneSplicer [0-21]
Branch Points [0-100]
Mutated Sequence
Alamut Visual Plus v.1.6.1 by SOPHiA GENETICS

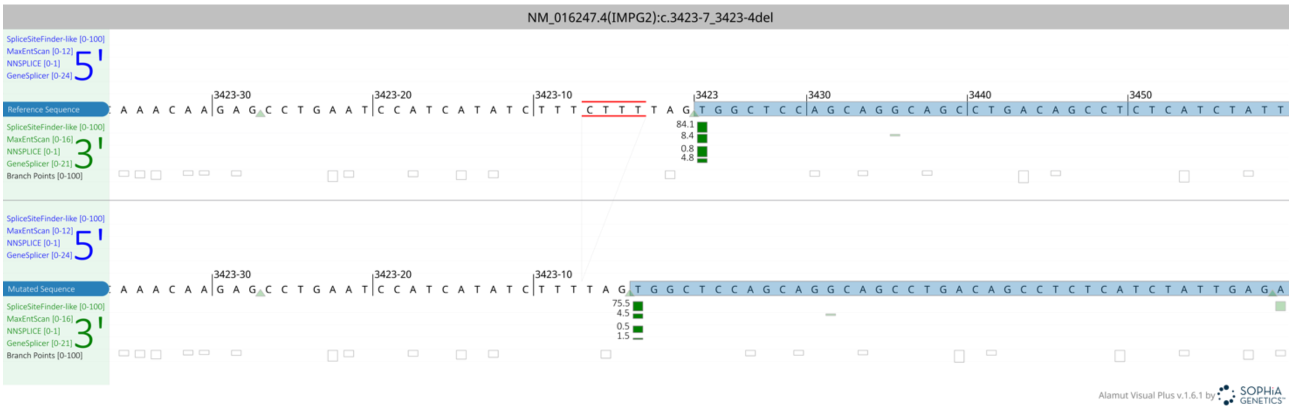
NM_016247.4(IMPG2):c.3423-7_3423-4del
Reference Sequence
Mutated Sequence
Alamut Visual Plus v.1.6.1 by SOPHiA GENETICS

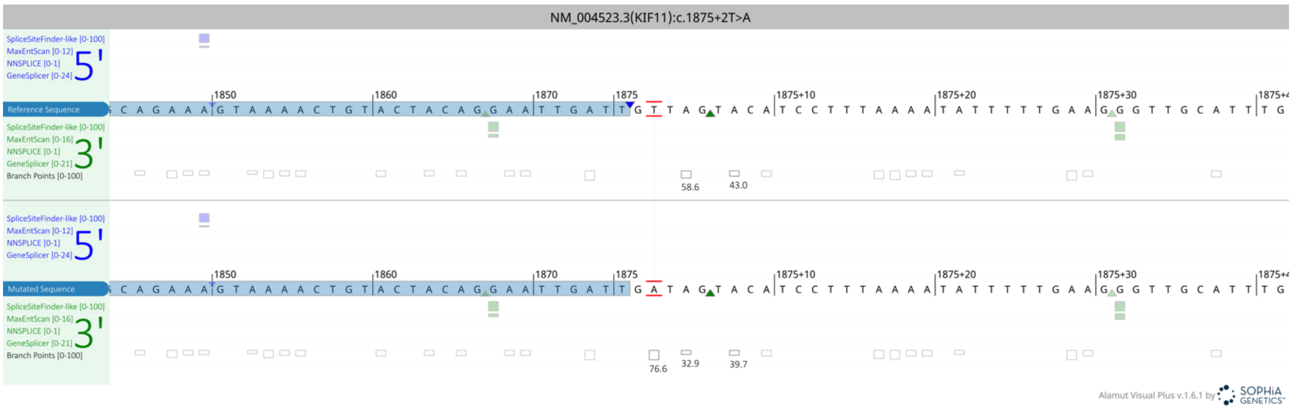
NM_004523.3(KIF11):c.1875+2T>A
Reference Sequence
Mutated Sequence
Alamut Visual Plus v.1.6.1 by SOPHiA GENETICS

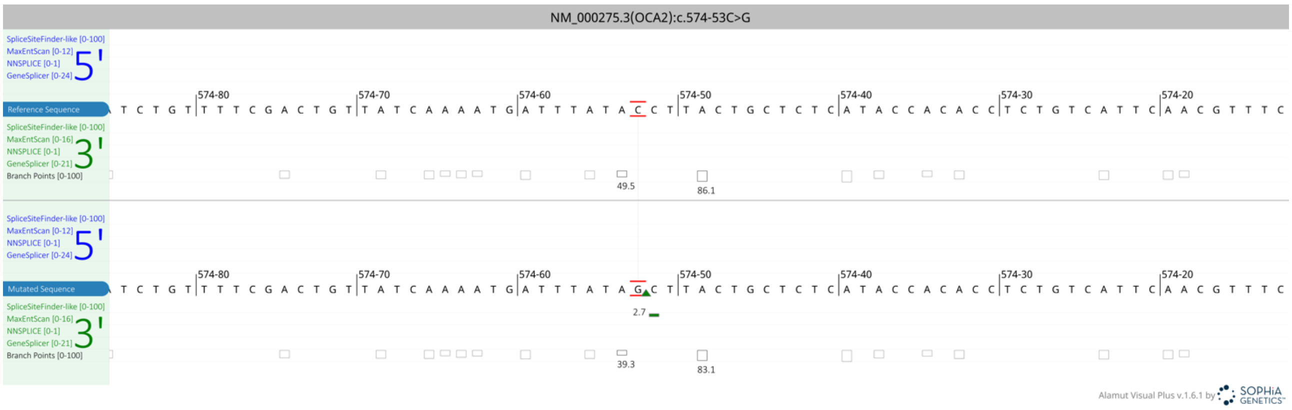
NM_000275.3(OCA2):c.574-53C>G
Reference Sequence
Mutated Sequence
Alamut Visual Plus v.1.6.1 by SOPHiA GENETICS

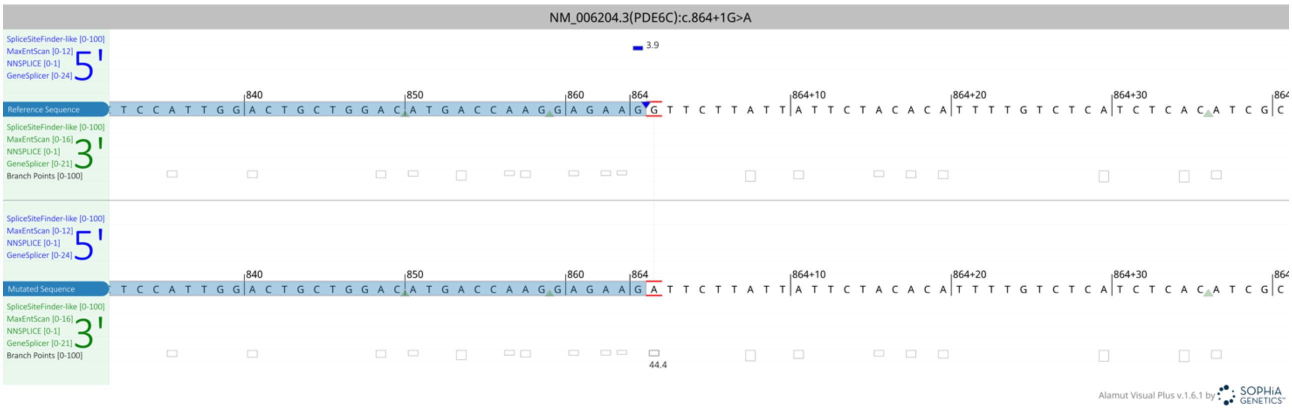
NM_006204.3(PDE6C):c.864+1G>A
Reference Sequence
Mutated Sequence
Alamut Visual Plus v.1.6.1 by SOPHiA GENETICS

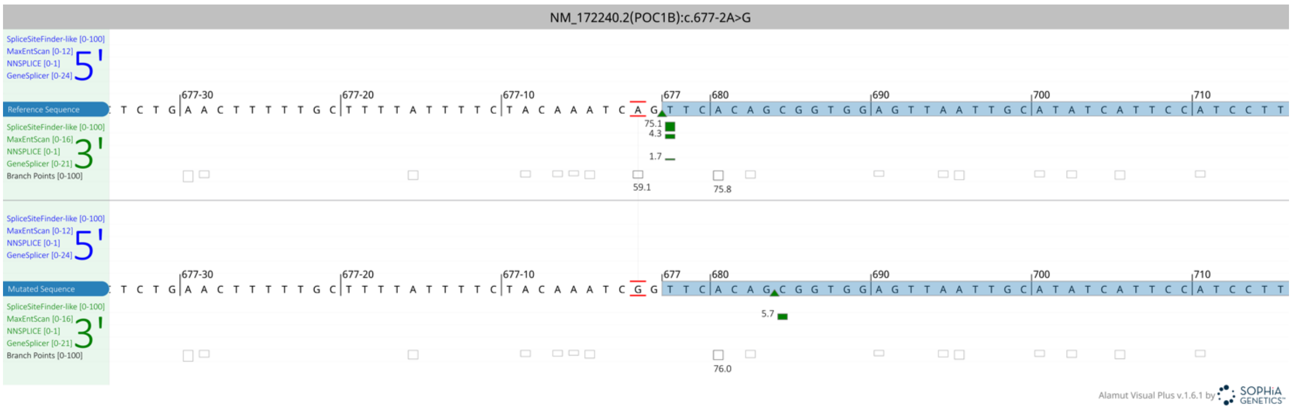
NM_172240.2(POC1B):c.677-2A>G
Reference Sequence
Mutated Sequence
SpliceSiteFinder-like [0-100]
MaxEntScan [0-12]
NNSPLICE [0-1]
GeneSplicer [0-24]
MaxEntScan [0-16]
GeneSplicer [0-21]
Branch Points [0-100]
Alamut Visual Plus v.1.6.1 by SOPHiA GENETICS

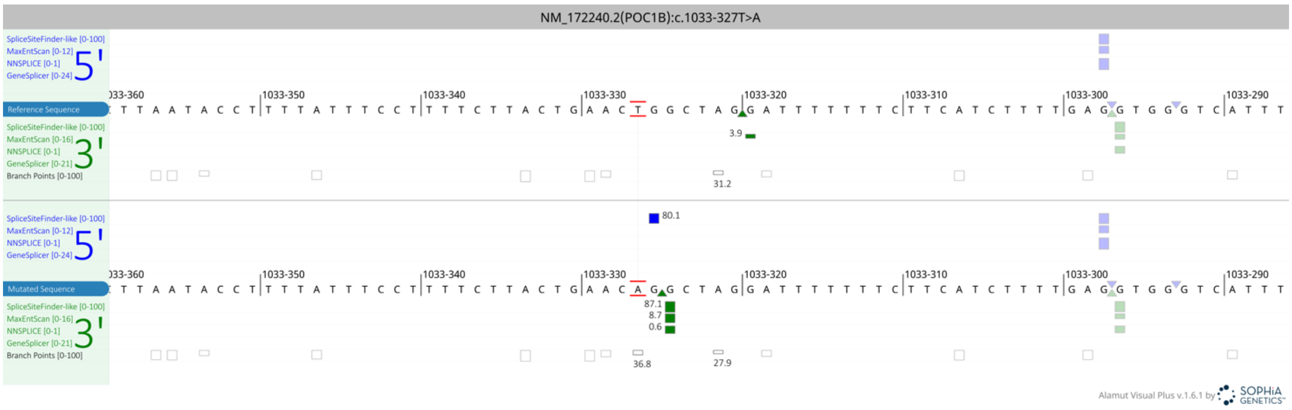
NM_172240.2(POC1B):c.1033-327T>A
Reference Sequence
Mutated Sequence
SpliceSiteFinder-like [0-100]
MaxEntScan [0-12]
NNSPLICE [0-1]
GeneSplicer [0-24]
MaxEntScan [0-16]
GeneSplicer [0-21]
Branch Points [0-100]
Alamut Visual Plus v.1.6.1 by SOPHiA GENETICS

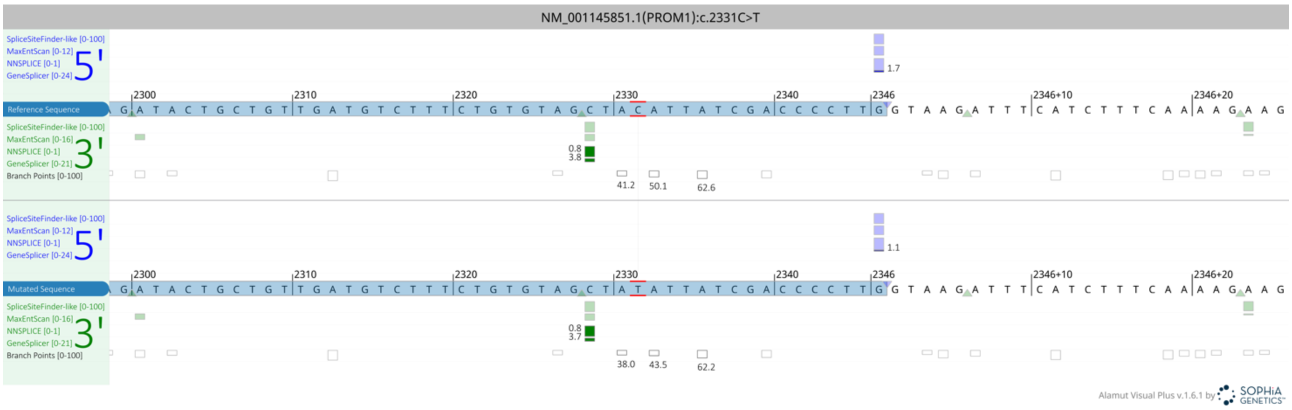
NM_001145851.1(PROM1):c.2331C>T
Reference Sequence
Mutated Sequence
SpliceSiteFinder-like [0-100]
MaxEntScan [0-12]
NNSPLICE [0-1]
GeneSplicer [0-24]
MaxEntScan [0-16]
GeneSplicer [0-21]
Branch Points [0-100]
Alamut Visual Plus v.1.6.1 by SOPHiA GENETICS

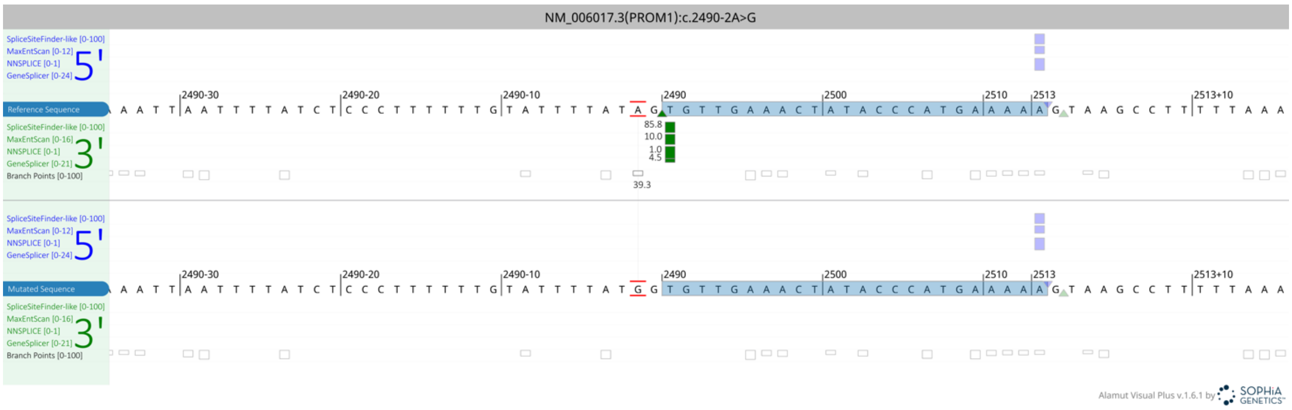
NM_006017.3(PROM1):c.2490-2A>G
Reference Sequence
Mutated Sequence
SpliceSiteFinder-like [0-100]
MaxEntScan [0-12]
NNSPLICE [0-1]
GeneSplicer [0-24]
MaxEntScan [0-16]
GeneSplicer [0-21]
Branch Points [0-100]
Alamut Visual Plus v.1.6.1 by SOPHiA GENETICS

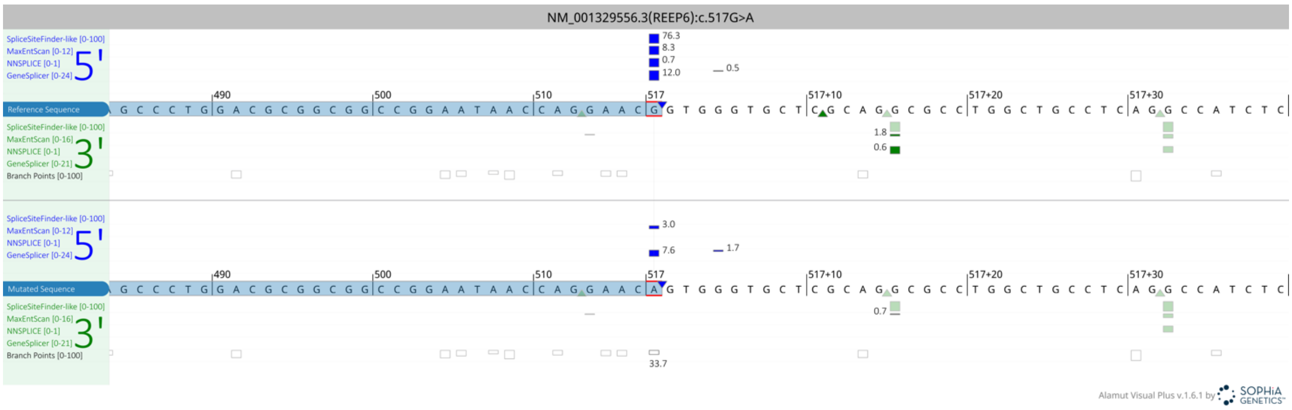
NM_001329556.3(REEP6):c.517G>A
SpliceSiteFinder-like [0-100]
MaxEntScan [0-12]
NNSPLICE [0-1]
GeneSplicer [0-24]
Reference Sequence
MaxEntScan [0-16]
GeneSplicer [0-21]
Branch Points [0-100]
Mutated Sequence
Alamut Visual Plus v.1.6.1 by SOPHiA GENETICS

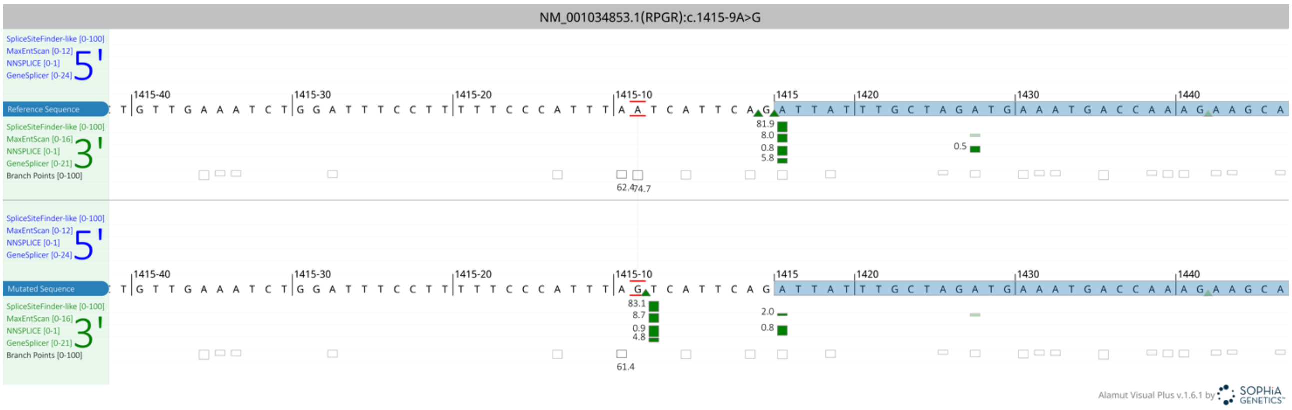
NM_001034853.1(RPGR):c.1415-9A>G
SpliceSiteFinder-like [0-100]
MaxEntScan [0-12]
NNSPLICE [0-1]
GeneSplicer [0-24]
Reference Sequence
MaxEntScan [0-16]
GeneSplicer [0-21]
Branch Points [0-100]
Mutated Sequence
Alamut Visual Plus v.1.6.1 by SOPHiA GENETICS

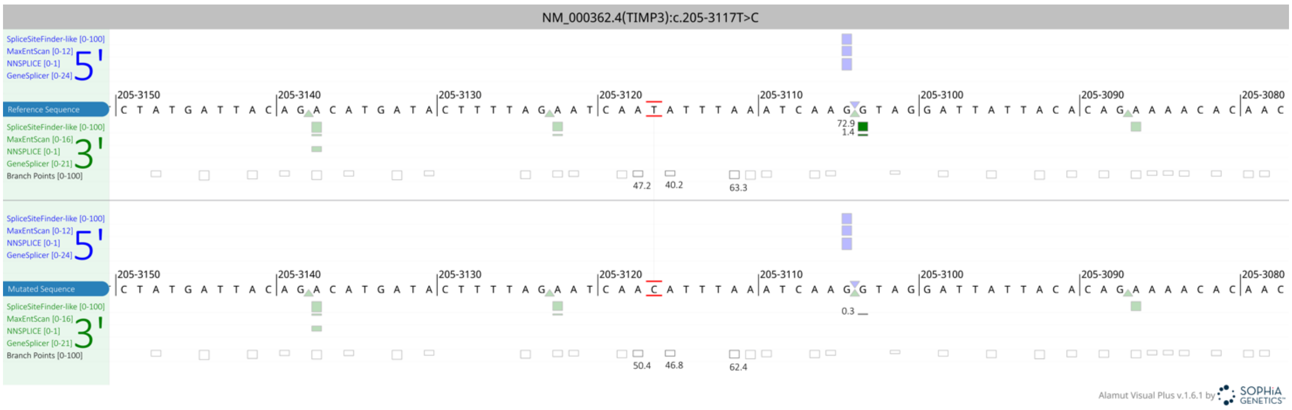
NM_000362.4(TIMP3):c.205-3117T>C
SpliceSiteFinder-like [0-100]
MaxEntScan [0-12]
NNSPLICE [0-1]
GeneSplicer [0-24]
Reference Sequence
MaxEntScan [0-16]
GeneSplicer [0-21]
Branch Points [0-100]
Mutated Sequence
Alamut Visual Plus v.1.6.1 by SOPHiA GENETICS

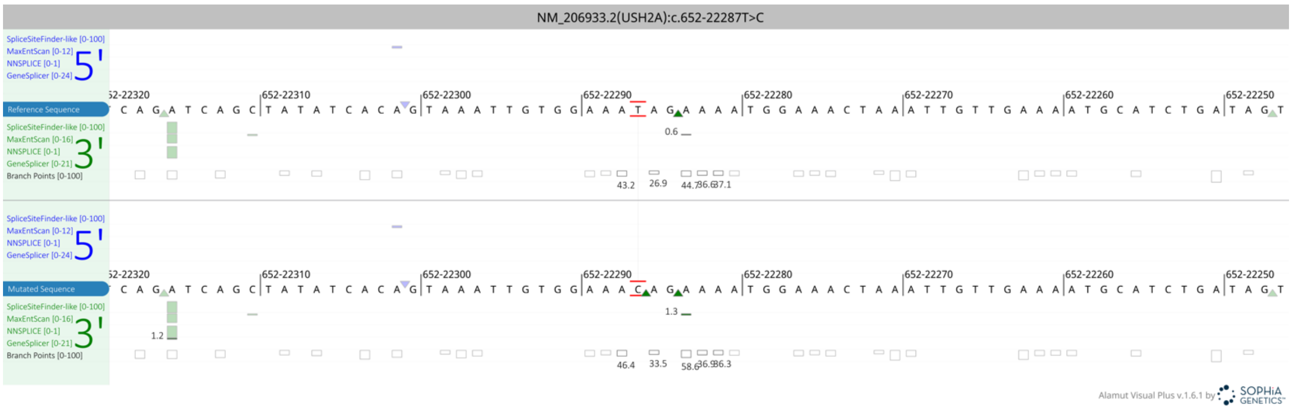
NM_206933.2(USH2A):c.652-22287T>C
SpliceSiteFinder-like [0-100]
MaxEntScan [0-12]
NNSPLICE [0-1]
GeneSplicer [0-24]
Reference Sequence
MaxEntScan [0-16]
GeneSplicer [0-21]
Branch Points [0-100]
Mutated Sequence
Alamut Visual Plus v.1.6.1 by SOPHiA GENETICS
