# Supplementary material for: Nanopore Deep Sequencing as a Tool to Characterize and Quantify Aberrant Splicing Caused by Variants in Inherited Retinal Dystrophy Genes
Source: Int J Mol Sci. 2024 Sep 3;25(17):9569. doi: 10.3390/ijms25179569 (PMC11395040; doi:10.3390/ijms25179569)

Figure S2. Minigene plasmids circular maps.

pcDNA3_RHO_ABCA4_int4-6_construct (12122 bp)

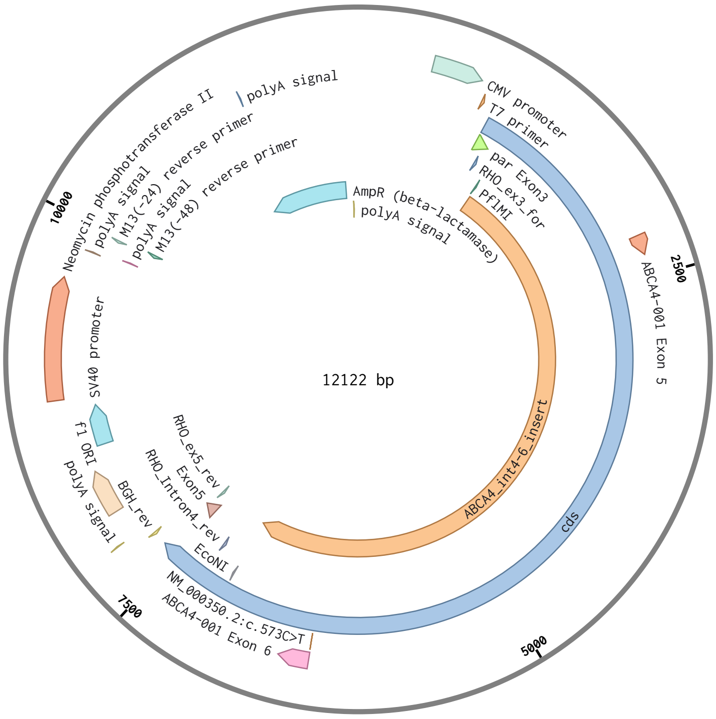

pcDNA3_RHO_ABCA4_int38-41_construct (10609 bp)

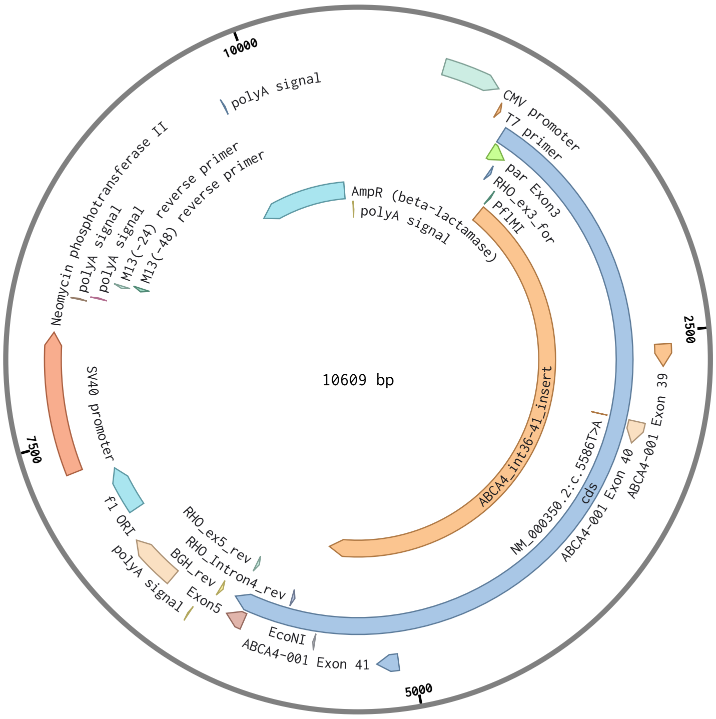

pcDNA3_RHO_ATF6_int8-9_construct (6686 bp)

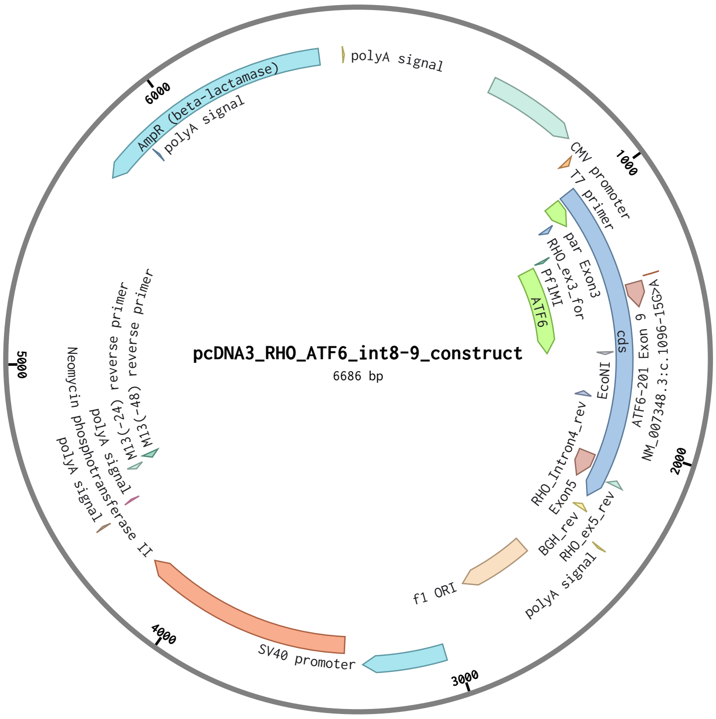

pcDNA3_ATF6_ex1-2-9_construct (7353 bp)

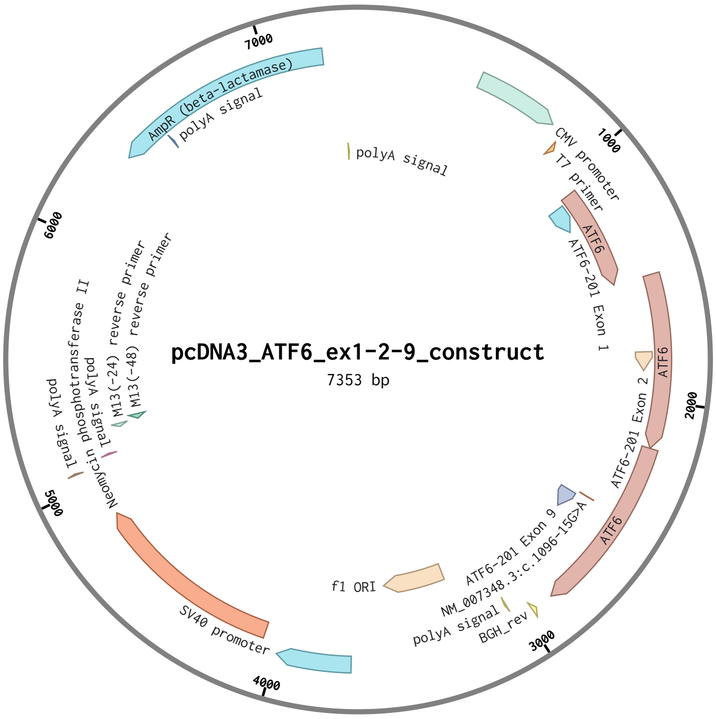

pcDNA3_ATF6_ex1-2-13_construct (7352 bp)

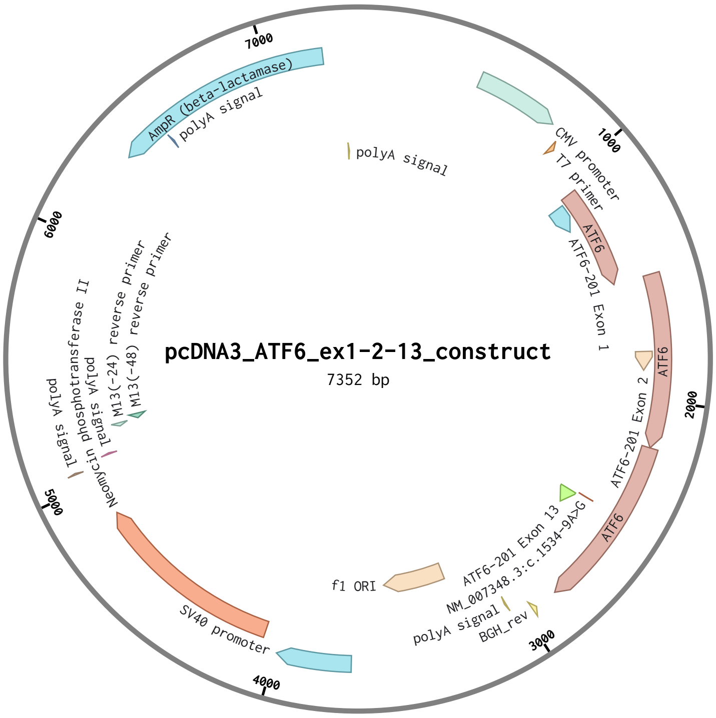

pcDNA3_RHO_CACNA1F_int14-18_construct (8751 bp)

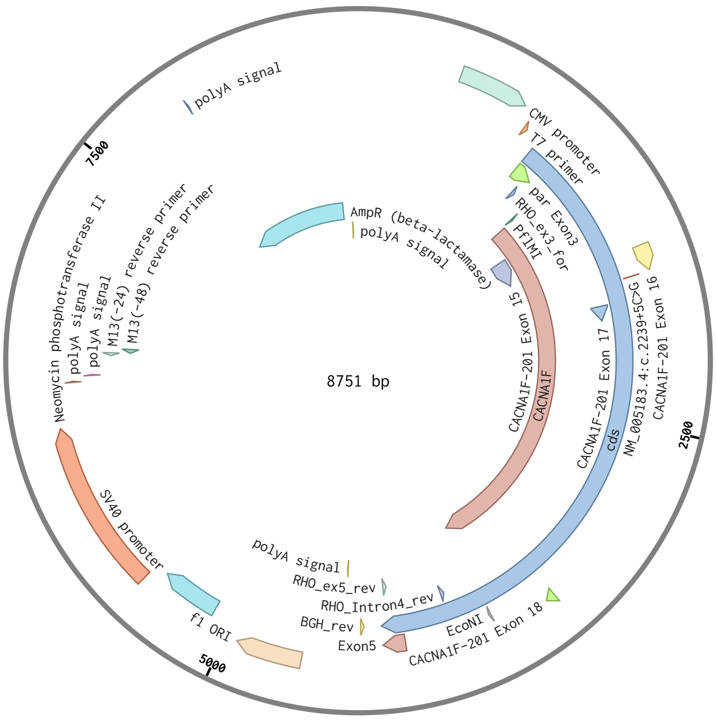

pcDNA3_RHO_CHM_int9-11_construct (7965 bp)

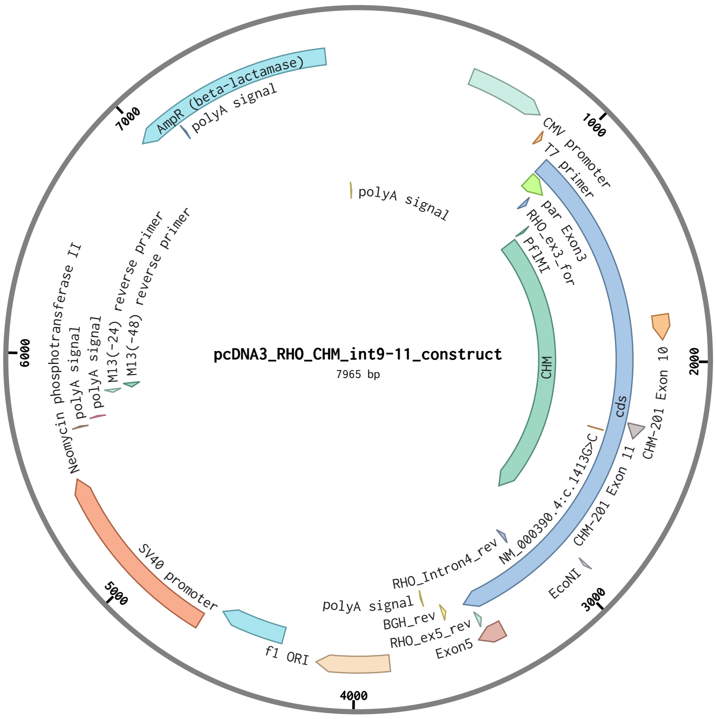

pcDNA3_FZD4_ex1-2 (9828 bp)

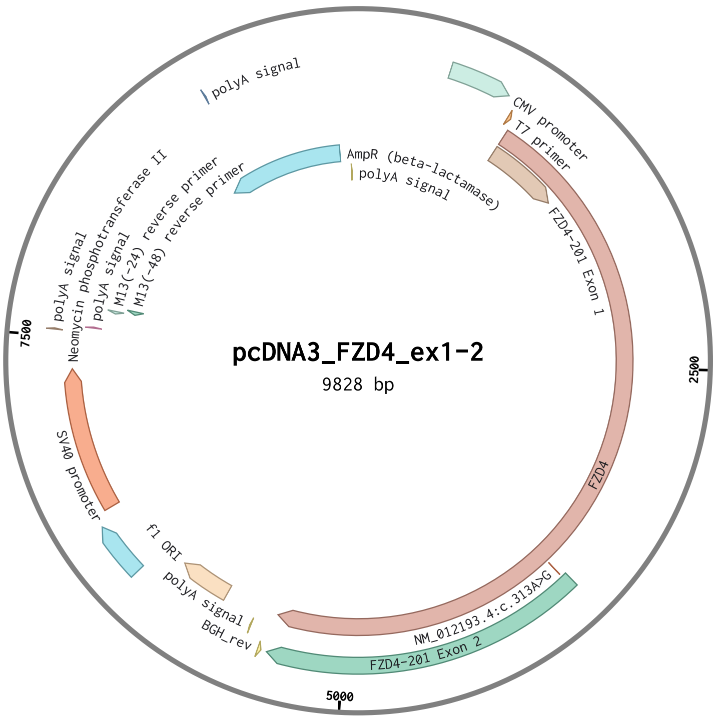

pcDNA3_RHO_IMPG2_int15-18 (9158 bp)

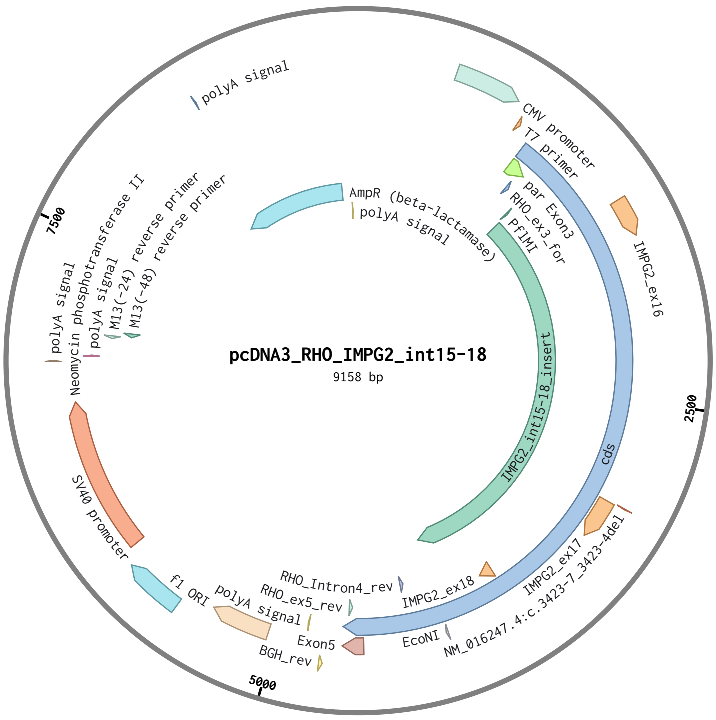

pcDNA3_RHO_IMPG2_int16-17 (7769 bp)

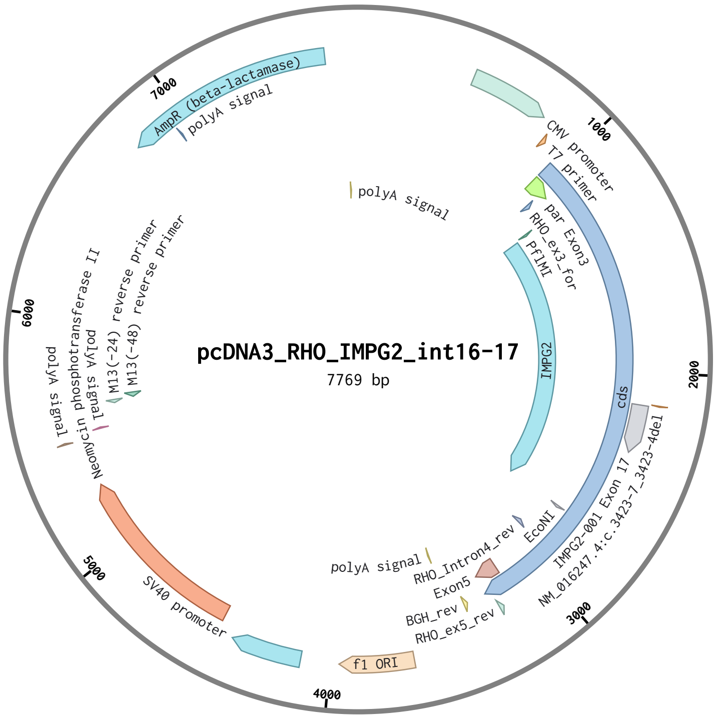

pcDNA3_RHO_OCA2_int5-7 (11516 bp)

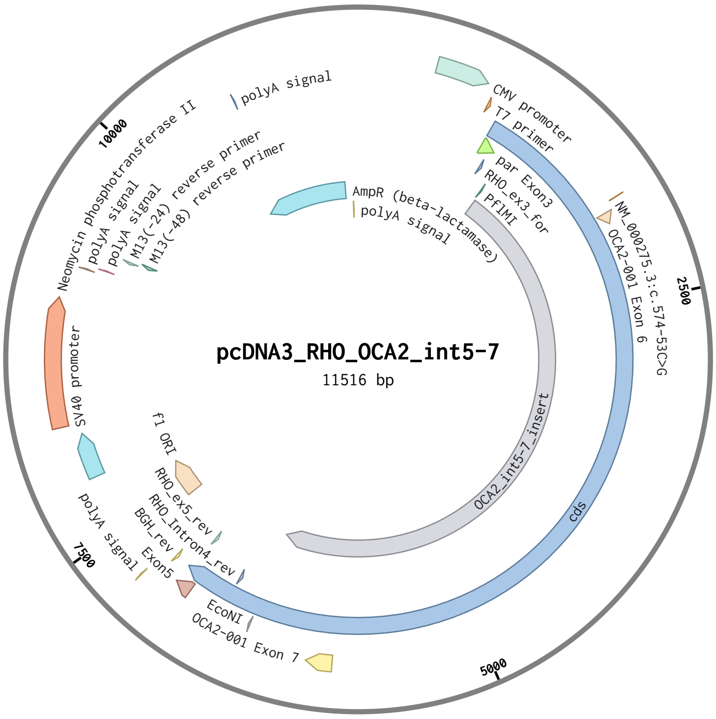

pcDNA3_RHO_PDE6C_int3-4_construct (7922 bp)

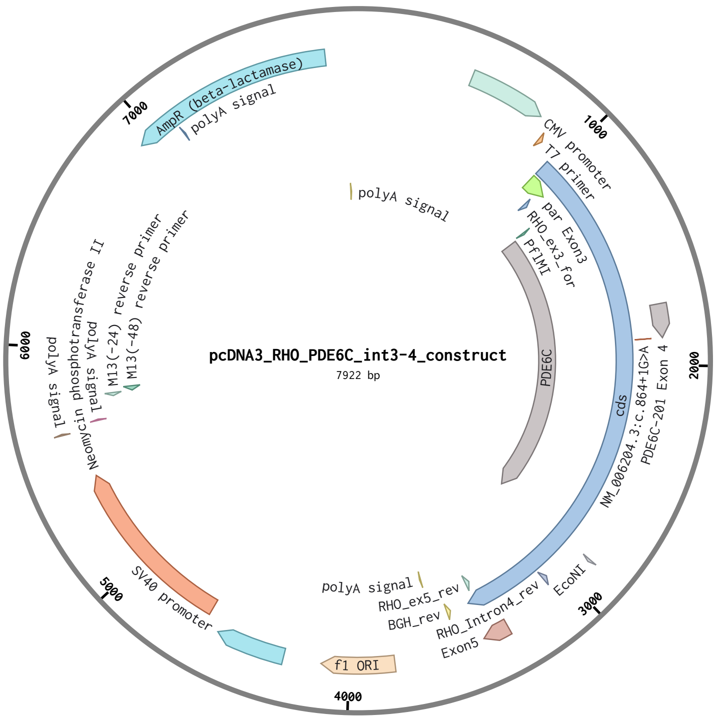

pcDNA3_RHO_POC1B_int6-7_construct (8217 bp)

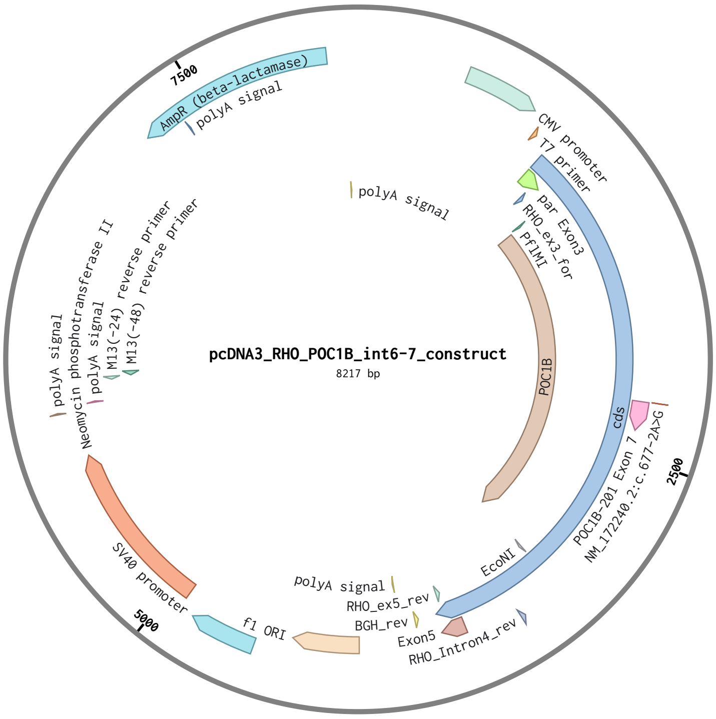

pcDNA3_RHO_POC1B_int9-10_construct (8460 bp)

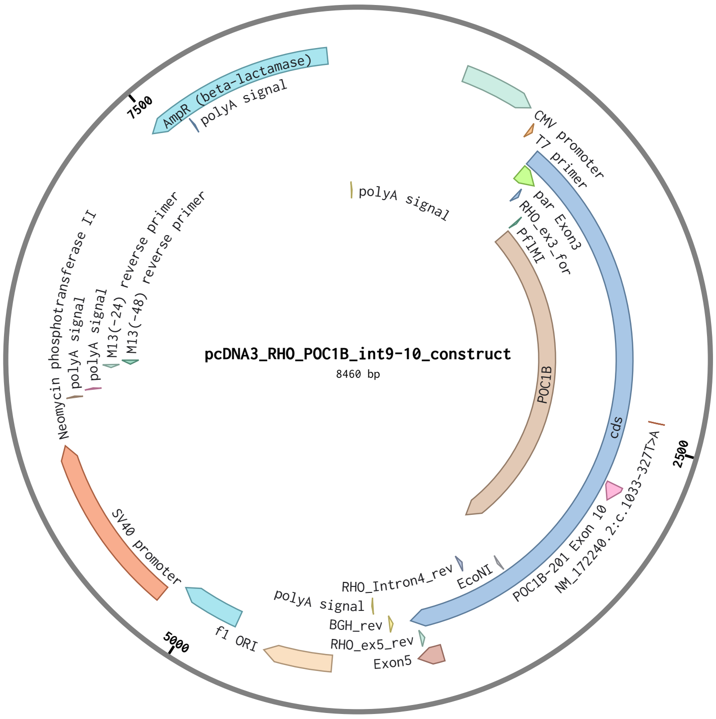

pcDNA3_RHO_PROM1_int20-23_construct (10963 bp)

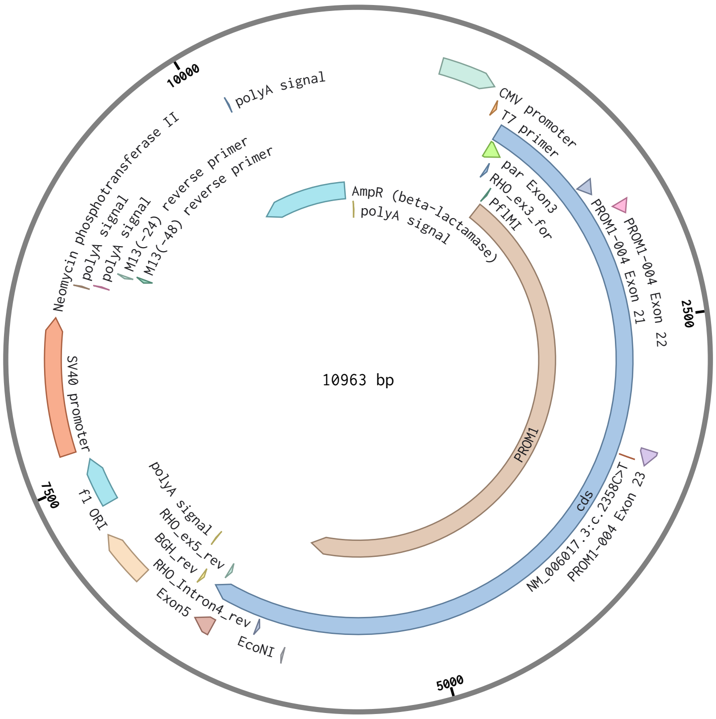

pcDNA3_RHO_PROM1_int23-26_construct (8301 bp)

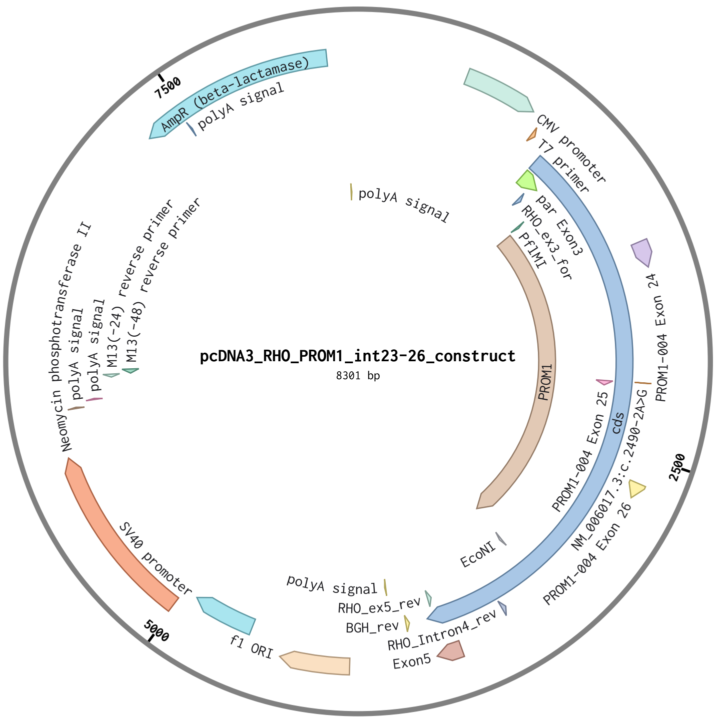

pcDNA3_RHO_REEP6_int1-5 (8217 bp)

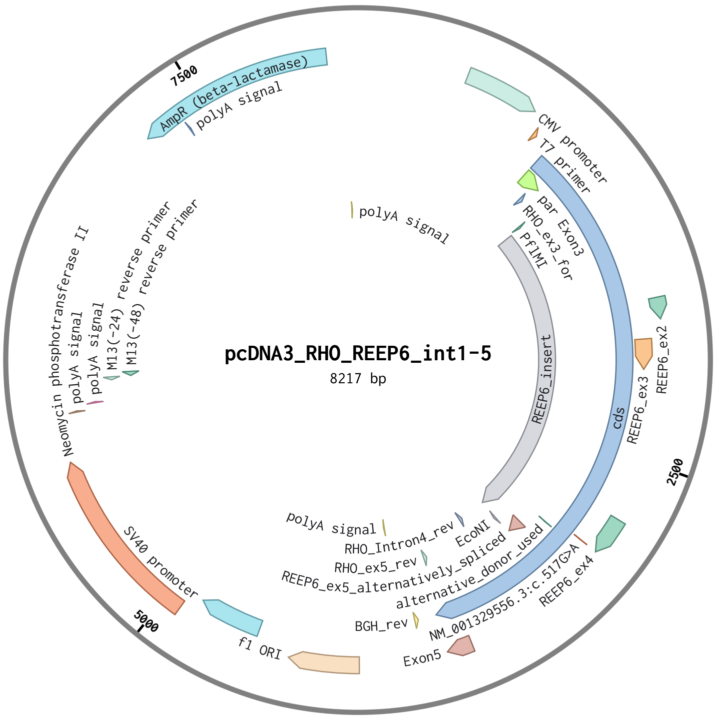

pcDNA3_RHO_RPGR_int10-13_construct (13308 bp)

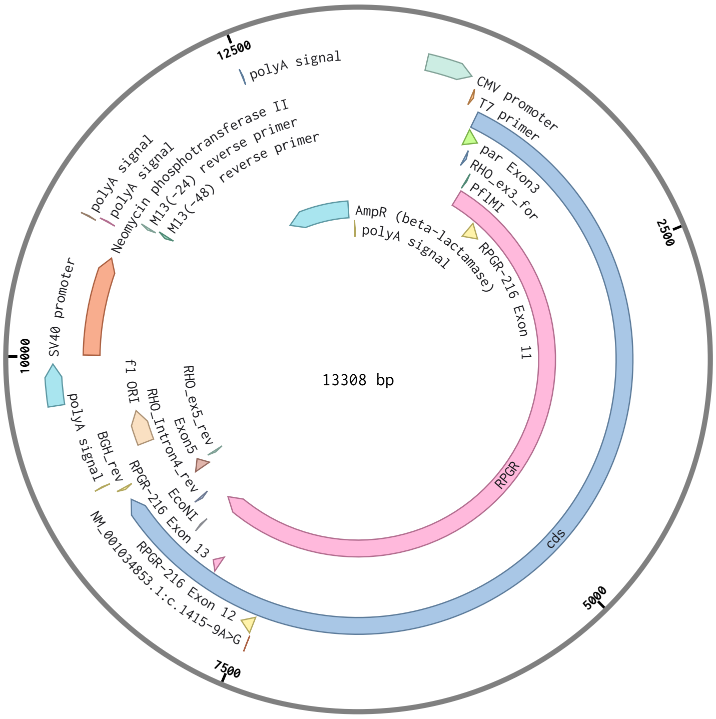

pcDNA3_RHO_TIMP3_int1-3_construct (14931 bp)

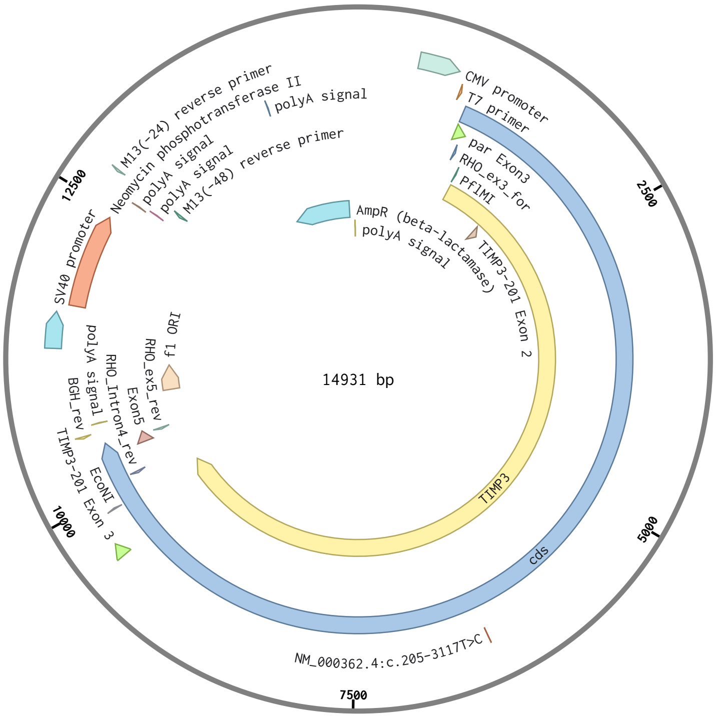

pcDNA3_RHO_USH2A_int3 (11044 bp)

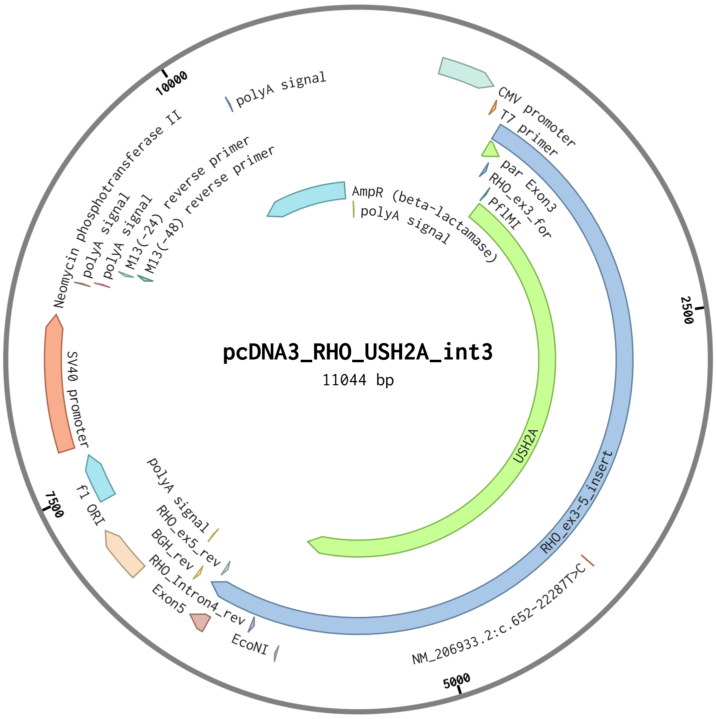

Supplement: Supplementary file 1 [file ijms-25-09569-s001.zip › Supplementary_materials/Supplementary_Figure_S2.pdf]
